# Biodistribution of cerium dioxide and titanium dioxide nanomaterials in rats after single and repeated inhalation exposures

**DOI:** 10.1186/s12989-024-00588-4

**Published:** 2024-08-14

**Authors:** Ilse Gosens, Jordi Minnema, A. John F. Boere, Evert Duistermaat, Paul Fokkens, Janja Vidmar, Katrin Löschner, Bas Bokkers, Anna L. Costa, Ruud J.B. Peters, Christiaan Delmaar, Flemming R. Cassee

**Affiliations:** 1 National Institute for Public Health and the Environment, PO box 1, Bilthoven, MA, 3720 The Netherlands; 2 National Food Institute, Technical University of Denmark, Kongens Lyngby, Denmark; 3 National Research Council, Institute of Science and Technology for Ceramics, Faenza, Italy; 4 Wageningen Food Safety Research, Wageningen, The Netherlands; 5 Institute for Risk Assessment Studies, Utrecht University, Utrecht, The Netherlands

**Keywords:** Inhalation exposure, In vivo, Poorly soluble nanoparticles, Cerium dioxide NM-212, Titanium dioxide NM-105, Tissue distribution, Lung clearance, Toxicity, Physiologically-based kinetic modelling

## Abstract

**Background:**

Physiologically based kinetic models facilitate the safety assessment of inhaled engineered nanomaterials (ENMs). To develop these models, high quality datasets on well-characterized ENMs are needed. However, there are at present, several data gaps in the systemic availability of poorly soluble particles after inhalation. The aim of the present study was therefore to acquire two comparable datasets to parametrize a physiologically-based kinetic model.

**Method:**

Rats were exposed to cerium dioxide (CeO_2_, 28.4 ± 10.4 nm) and titanium dioxide (TiO_2,_ 21.6 ± 1.5 nm) ENMs in a single nose-only exposure to 20 mg/m^3^ or a repeated exposure of 2 × 5 days to 5 mg/m^3^. Different dose levels were obtained by varying the exposure time for 30 min, 2 or 6 h per day. The content of cerium or titanium in three compartments of the lung (tissue, epithelial lining fluid and freely moving cells), mediastinal lymph nodes, liver, spleen, kidney, blood and excreta was measured by Inductively Coupled Plasma-Mass Spectrometry (ICP-MS) at various time points post-exposure. As biodistribution is best studied at sub-toxic dose levels, lactate dehydrogenase (LDH), total protein, total cell numbers and differential cell counts were determined in bronchoalveolar lavage fluid (BALF).

**Results:**

Although similar lung deposited doses were obtained for both materials, exposure to CeO_2_ induced persistent inflammation indicated by neutrophil granulocytes influx and exhibited an increased lung elimination half-time, while exposure to TiO_2_ did not. The lavaged lung tissue contained the highest metal concentration compared to the lavage fluid and cells in the lavage fluid for both materials. Increased cerium concentrations above control levels in secondary organs such as lymph nodes, liver, spleen, kidney, urine and faeces were detected, while for titanium this was found in lymph nodes and liver after repeated exposure and in blood and faeces after a single exposure. Conclusion: We have provided insight in the distribution kinetics of these two ENMs based on experimental data and modelling. The study design allows extrapolation at different dose-levels and study durations. Despite equal dose levels of both ENMs, we observed different distribution patterns, that, in part may be explained by subtle differences in biological responses in the lung.

**Supplementary Information:**

The online version contains supplementary material available at 10.1186/s12989-024-00588-4.

## Background

Exposure to low concentrations of poorly soluble engineered nanomaterials (ENMs) over a prolonged period of time may increase the risk for adverse health effects due to potential accumulation of these materials in tissues and organs. Nanomaterials seem to translocate from the site of intake to the blood, reach secondary organs and may accumulate there [1–5]. A better understanding of the process of distribution through the body after deposition in the lung, the potential for accumulation in secondary organs and excretion from the body will help to assess their safety.

To accommodate the need for safety assessment of poorly soluble nanomaterials, non-animal models have been developed in EU funded project PATROLS (Physiologically anchored tools for nanomaterials risk assessment, www.patrols-h2020.eu). For example, physiologically-based kinetic (PBK) models are mathematical models that can help to understand the biodistribution of chemicals including ENMs [6]. For ENMs, these models are still in their infancy. However, a promising model has been developed by Li et al. [7]. This model predicts the distribution and accumulation of cerium dioxide (CeO_2_) in rat tissues. One way to further develop these PBK models for ENMs is to improve their parametrisation by using comparable detailed high quality in vivo data. This in term can also be used to optimize inhalation study designs following for instance OECD test guideline 412 and 413, as well as assess which secondary organs can be a target for inhaled ENMs and what would be a desired dose range for in vitro testing.

A literature review identified two ENMs [8], i.e. CeO_2_ NM-212 and TiO_2_ NM-105, as promising candidates for PBK model parameterisation. Detailed data on these ENMs have already been generated on the physico-chemical [9, 10] and toxicological properties after inhalation [11–13] as well as after intratracheal instillation [14]. While some biodistribution and lung burden data was already available for these materials, some important details were lacking. For CeO_2_ NM-212, some researchers have already reported detailed lung and lymph nodes burdens sampled in the post-exposure period [15], and have also addressed lung clearance by measuring lung burdens during the exposure [13]. However, information on excretion of cerium from secondary organs after the exposure is stopped for periods over 72 h is lacking, while a period of weeks would be important based on the expected slow excretion of poorly soluble materials. Liver burdens [12] as well as more comprehensive secondary organ distribution have been determined in spleen, kidney, brain, testis and epididymis as well as olfactory bulbs, small intestine, bone and bone marrow [13, 16]. However, there is a lack of data on cerium levels in excreta which are needed to estimate absorption and excretions rates from the body. Such data are informative to assess the absorption from the lung as well as the relative contribution of extrapulmonary tissue deposition via the oral route following inhalation exposure of particles (e.g., via activity of the mucociliary escalator mediated by macrophages) [16]. It is also not known whether the dose delivered to secondary organs is linearly dependent on the inhaled dose levels.

For TiO_2_, a quantitative biokinetic study has been performed using 20 nm radioactively labelled [48 V]-TiO_2_ [17]. This included different compartments of the lung by determining the amount of titanium in lavaged and non-lavaged lung tissue, the bronchoalveolar lavage fluid (BALF) as well as the cells in the BALF that have phagocytic activity. A similar compartmentalisation of the lung by lavage has been applied here, but for a (non-radioactively labelled) TiO_2_ ENM with a similar primary particle size of 20 nm as well as for a CeO_2_ ENM.

TiO_2_ NM-105, which is very similar to TiO_2_ P25, has been widely studied. Toxicological effects in the lung, such as lung cell damage and inflammation have been previously described, occasionally together with the associated lung burdens [14, 18–20]. After a 5 day inhalation of 0.1 mg/m^3^ of a similar type of TiO_2_ ENM (30% rutile/70% anatase with a primary particle size of 20–30 nm), titanium was detectable in lung and lymph nodes, but not in liver, spleen, kidney and brain [21] either 3 days or 14 days after the last exposure. Similar as for CeO_2_, measurements on titanium levels in secondary organs and excreta beyond a time period of 14 days after ceasing the inhalation exposure are lacking.

Here, we have generated datasets for both CeO_2_ and TiO_2_ ENMs, both following the same inhalation exposure study design for which we hypothesized that these will follow a very similar biodistribution pattern upon the same exposure levels and duration. This will allow modelling differences in translocation at different deposited doses (low, mid and high) in the lung, while obtaining information on the different compartments in the lung. We have chosen the dose levels such that we did not expect damage to the alveolar epithelium-blood barrier based on cellular damage markers, total cell counts and differential cell counts in the bronchoalveolar lavage for at least two out of the three applied exposure regimes. Finally, single and repeated exposures with similar aerosol generation conditions were studied for both ENMs. This approach provides insight into how measured ENM concentrations in the lung (divided in different compartments as performed previously [17]), mediastinal lymph nodes, liver, kidney and spleen scale with the duration and frequency of exposure.

## Results

### Choices in study design following pilot studies

Prior to exposing rats in the main biodistribution study, two pilot studies were performed with CeO_2_ NM-212 and TiO_2_ NM-105 ENMs. To set the exposure concentration in the pilot study, multiple-path particle dosimetry (MPPD V3.14) modelling was used to estimate the deposited fractions in the different lung regions (for details see additional file 1). The mass median aerodynamic diameter (MMAD) was assumed around 1.4 μm for both ENMs based on earlier reported values [11, 12, 18, 19]. Then, the pilot study (for aerosol characteristics see additional file 1, Table S1) was performed to check whether cerium could indeed be detected in the different lung compartments (bronchoalveolar lavage (BAL) fluid, cells collected by BAL and lavaged lung tissue) and liver as predicted, 18 h after a single nose-only exposure (Additional file 1, Table S2). A similar pilot study set-up was used for a single nose-only exposure to TiO_2_ to determine detection of titanium (Additional file 1, Table S3). Following TiO_2_ exposure, the titanium content in the liver was not above control levels at 18 h after exposure.

Subsequently, toxicity markers were determined in the BAL fluid to assess whether a single exposure to a target concentration of 20 mg/m^3^ would lead to acute lung toxicity. Cell damage markers lactate dehydrogenase (LDH) and protein as well as total cell counts, percentage macrophages, percentage neutrophils were determined in the bronchoalveolar lavage fluid (BALF) after exposure to CeO_2_ (Additional file 1, Table S4) and TiO_2_ (Additional file 1, Table S5). There was no change in cell damage and lung inflammation (percentage of macrophages or influx of neutrophils) due to the exposure to CeO_2_ or TiO_2_ compared to the controls. Particulate structures were identified inside macrophages in exposed animals, but not in control animals.

In absence of an acute cellular damage or strong pulmonary inflammatory response, we decided not to make any adaptations to the exposure schedule for the single exposures in the main experiment. Since we also wanted to apply repeated exposures in the main study, we reduced the exposure concentration to reach a comparable lung burden after a 2 times 5 day exposure of 6 h per day compared to a single 6 h exposure.

### Exposure characteristics of the main study

During the entire exposure period of the main studies, the aerosols generated from dry powders of CeO_2_ NM-212 (Table 1) and TiO_2_ NM-105 (Table 2) have been characterized. The actual exposure concentrations were close to the target concentrations. The MMAD was determined shortly after the exposure of the animals was finished, was close to the assumed MMAD for modelling and within the recommended MMAD of ≤ 2 μm with a GSD of 1–3 [22]. Also, the MMAD and geometric standard deviation for both CeO_2_ and TiO_2_ aerosols were very similar per exposure schedule as well as to each other, around 1 μm.

**Table 1:** Aerosol parameters of CeO_2_ NM-212

Nominal particle size (nm)	28.4 ± 10.4					
Estimated effective density (g/cm^3^)	1.5–2.4					
Exposure schedule	1 day*			2 × 5 days#		
Target concentration (mg/m^3^)	20			5		
Gravimetric concentration (mg/m^3^) ± sd	20.9	±	1.60	5.07	±	0.12
Particle number concentration (p/cc) ± sd	5.5E + 05	±	1.10E + 05	7.1E + 04	±	6.3E + 03
MMAD (µm) ± GSD	1.05	±	1.66	1.09	±	1.76
CMD (nm) ± gsd	152	±	1.85	154	±	1.86

* relative humidity of the test atmosphere was 46.2–61.4%RH and the temperature was 22.3–24.2 °C

# relative humidity of the test atmosphere was 47.3–60.7%RH and temperature was 22.1–24.4 °C

**Table 2:** Aerosol parameters of TiO_2_NM-105

Nominal particle size (nm)	21.6 ± 1.5					
Estimated effective density (g/cm^3^)	4.3					
Exposure schedule	1 day*			2 × 5 days#		
Target concentration (mg/m^3^)	20			5		
Gravimetric concentration (mg/m^3^) ± sd	19.1	±	1.0	5.02	±	0.15
Particle number concentration (p/cc) ± sd	1.3E + 05	±	1.32E + 04	4.1E + 04	±	6.0E + 03
MMAD (µm) ± gsd	0.99	±	1.49	1.00	±	1.63
CMD (nm) ± gsd	233	±	1.72	226	±	1.80

* relative humidity of the test atmosphere was 48.1–59.0%RH and the temperature was 22.5–24.4 °C

# relative humidity of the test atmosphere was 48.9–62.7%RH and the temperature was 22.6–24.5 °C

### Cerium and titanium lung concentrations per compartment

**Fig. 1:**
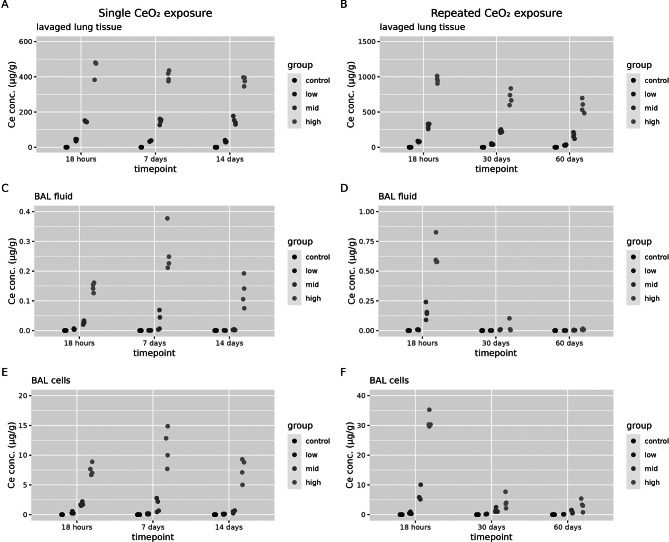
Cerium concentrations in lung compartments. These were assessed 18 h, 7 days and 14 days after a single (1 day) CeO_2_ exposure and at 18 h, 30 days or 60 days after a repeated (2 × 5 days) CeO_2_ exposure in control animals and low, mid and high exposure groups (*n* = 4 per group) in (**A**) lavage lung tissue after a single exposure, (**B**) lavage lung tissue after a repeated exposure, (**C**) BAL fluid after a single exposure (**D**) BAL fluid after a repeated exposure, (**E**) BAL cells after a single exposure and (**F**) BAL cells after a repeated exposure. Cerium concentrations in lavage lung tissue are expressed as µg/g of dry weight, and in BAL fluid and BAL cells as µg/g of wet weight

Cerium concentrations were determined in different lung compartments of the lavaged lung tissue, BAL cells and BAL fluid (Fig. 1). A dose-dependent increase in cerium concentration in the lavaged lung compartment was observed after a single and repeated exposure, and at all time points assessed after exposure (see additional file 2, Table S1 for statistical analysis). The cerium in the lavaged lung tissue is representative of the fraction of the cerium that resides in the tissue plus some of the phagocytic cells that remain in the tissue after lavage. After a single exposure, in the BAL fluid and BAL cell compartment, there is only a significant increase in cerium concentrations at the high dose (and sometimes mid dose) levels compared to controls. The cerium detected in the BAL cells can (most likely) be attributed to cerium taken up by phagocytic cells such as macrophages. The BAL fluid contained the lowest cerium concentrations and is considered to be representative of particles that reside freely available on the alveolar surface and can therefore be washed away during a bronchoalveolar lavage. After repeated exposure, at timepoints 30 and 60 days after exposure, there is no longer a difference between cerium concentrations in controls and exposed rats in the BAL fluid compartment, while there is still a dose-dependent increase compared to controls in the lavaged lung tissue and BAL cell compartments.

**Fig. 2:**
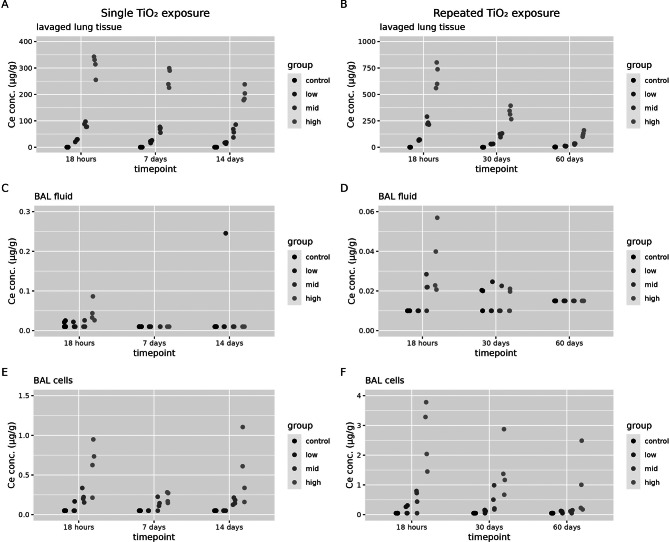
Titanium concentrations in lung compartments. These were assessed 18 h, 7 days and 14 days after a single (1 day) TiO_2_ exposure. Or at 18 h, 30 days or 60 days after a repeated (2 × 5 days) TiO_2_ exposure in control animals and low, mid and high exposure groups (*n* = 4 per group) in (**A**) lavage lung tissue after a single exposure, (**B**) lavage lung tissue after a repeated exposure, (**C**) BAL fluid after a single exposure (**D**) BAL fluid after a repeated exposure, (**E**) BAL cells after a single exposure and (**F**) BAL cells after a repeated exposure. Titanium concentrations in lavage lung tissue are expressed as µg/g of dry weight, and in BAL fluid and BAL cells as µg/g of wet weight

Titanium concentrations in different lung compartments of the lavaged lung tissue, BAL cell, BAL fluid are shown in Fig. 2. There is a dose-dependent increase in titanium concentrations in the lavaged lung compartment as well as in the BAL cells at all time points assessed after both single and repeated exposure (see additional file 3, Table S1 for statistical analysis). Over time, titanium levels in these compartments after both single and repeated exposure decline. After a single and repeated exposure in the BAL fluid compartment, there is only a dose-dependent increase in titanium concentrations 18 h after the exposure, but no longer at later timepoints (except for one high measured concentration in a single animal). This differs from cerium concentrations in the BAL fluid compartment as these still increase compared to controls at timepoints 7 and 14 days after a single exposure. Similar to the cerium measurements, the BAL fluid contains the lowest titanium concentrations.

### Elimination from the lung

For both inhalation experiments using CeO_2_ and TiO_2_ ENM aerosols, the clearance from the lung was determined by estimating the elimination half-times from the retained lung burdens in the post-exposure period (Table 3). The measured retained lung burdens were assessed with a similar calculation as described for the pilot study. The total lung burdens were calculated by adding the lavaged lung tissue dose (corrected for the total lung dry weight), BAL fluid dose (corrected for the total recovered BAL fluid volume and density of saline) and BAL cells dose at the different timepoints after exposure (corrected for the density of saline) (Additional file 3 for cerium tissue and excreta dose and additional file 4 for titanium tissue and excreta dose).

**Table 3:** Average measured total cerium and titanium retained lung burdens (*n* = 4) and estimated elimination half-time

Exposure group	Exposure time and concentration	Av. measured Ce lung burden ± sd (µg)			CeO_2_ half-time (days)	Av. measured Ti lung burden ± sd (µg)			TiO_2_ half-time (days)
		18 h	7 days	14 days		18 h	7 days	14 days	
Control, single	6 h on 1 day to clean air	0.01 ± 0.00	0.01 ± 0.001	0.01 ± 0.003	n.a.	0.27 ± 0.08	0.22 ± 0.01	0.25 ± 0.03	n.a.
Low, single	30 min on 1 day to 20 mg/m^3^	13 ± 1.7	12 ± 1.3	10 ± 0.9	40	9.4 ± 1.7	7.3 ± 1.1	6.1 ± 1.1	18
Mid, single	2 h on 1 day to 20 mg/m^3^	49 ± 4.1	48 ± 8.5	48 ± 6.9	n.d.	29 ± 3.0	23 ± 2.8	19 ± 5.1	20
High, single	6 h on 1 day to 20 mg/m^3^	145 ± 12	144 ± 7.5	143 ± 10	n.d.	91 ± 11	84 ± 5.0	68 ± 9.5	31
		**18 h**	**30 days**	**60 days**		**18 h**	**30 days**	**60 days**	
Control, single	6 h on 1 day to clean air	0.01 ± 0.001	0.01 ± 0.001	0.01 ± 0.004	n.a.	0.24 ± 0.01	0.34 ± 0.07	1.21 ± 0.19	n.a.
Low, repeated	30 min on 2 × 5 days to 5 mg/m^3^	26 ± 3.3	17 ± 2.6	13 ± 2.2	58	22 ± 2.1	10 ± 0.74	4.5 ± 0.76	25
Mid, repeated	2 h on 2 × 5 days to 5 mg/m^3^	110 ± 5.1	92 ± 8.4	70 ± 11	99	82 ± 4.3	41 ± 5.7	11 ± 2.0	20
High, repeated	6 h on 2 × 5 days to 5 mg/m^3^	351 ± 15	269 ± 32	239 ± 27	134	241 ± 30	120 ± 18	50 ± 12	26

n.a. not applicable for control group

n.d. could not be estimated due to no significant decrease over studied time period

For both CeO_2_ and TiO_2_ exposures, with increased exposure time (from 30 min to 2 h to 6 h), the total deposited lung dose increases (at t = 18 h). After the exposure period, with increased post-exposure sampling time, the lung burden decreases. A larger decrease is found for titanium compared to cerium. Subsequently, the estimated clearance from the lung after 30 min of a single exposure to 20 mg/m^3^ of CeO_2_ is slower (t_1/2_ of 40 days) compared to a similar exposure concentration and duration to TiO_2_ (t_1/2_ of 18 days) in the lowest exposure group. For CeO_2,_ after a single exposure, this could not be estimated with enough certainty for the middle and high exposure groups, since there is hardly any clearance from the lung over the 14-day time period, while there is clearance of titanium from the lung. After repeated exposure to CeO_2_, an increase in estimated elimination half-time with increasing dose levels is found from 58 to up to 134 days. The estimated elimination half-time after repeated exposure to TiO_2_ stays the same with increasing dose levels (t_1/2_ of 20–25 days).

### Distribution, relative translocation and elimination from the body

**Fig. 3:**
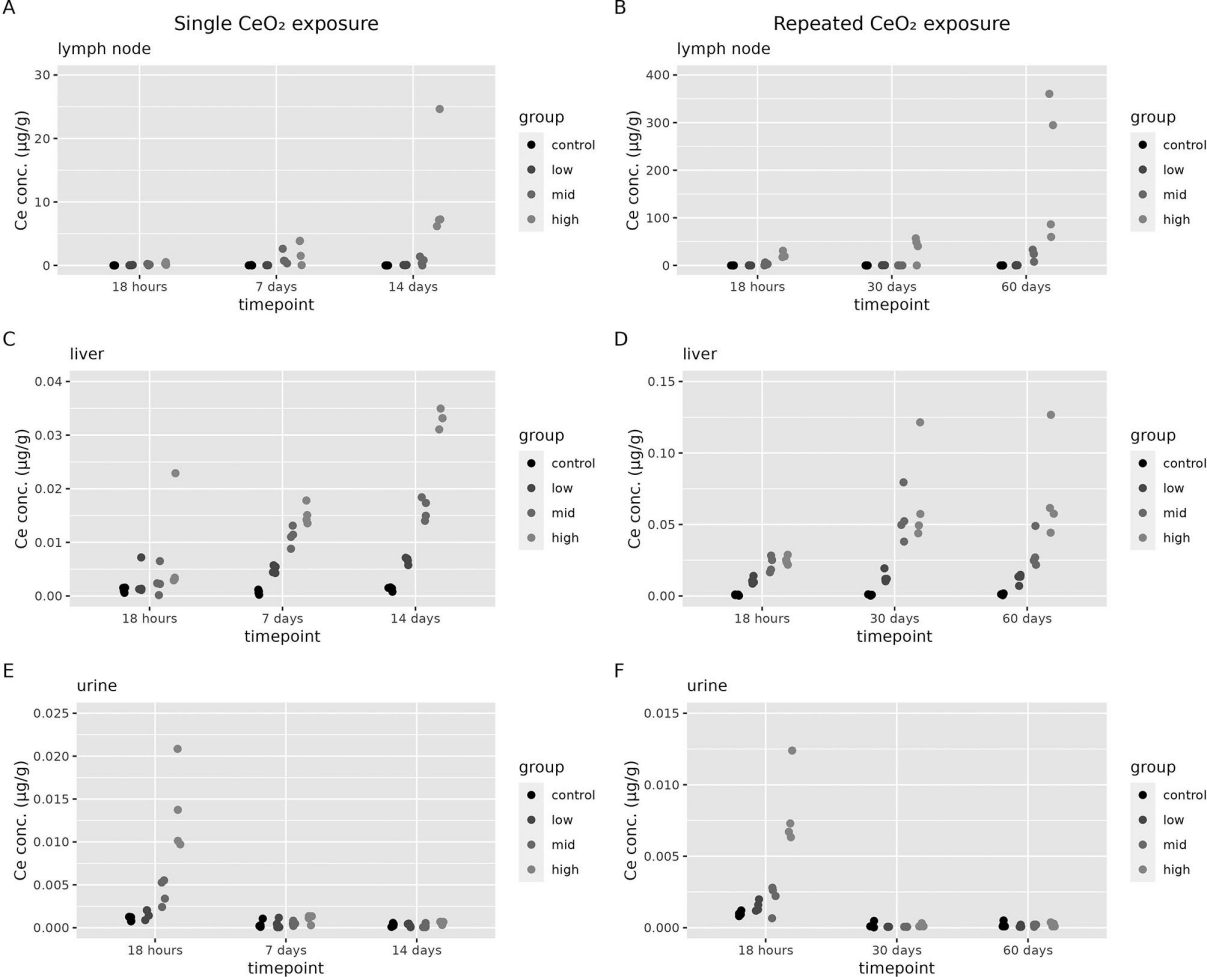
Biodistribution of cerium to secondary compartments. Cerium organ concentrations (in µg/g organ dry weight) and urine concentration (in µg/g) were assessed 18 h, 7 days and 14 days after a single (1 day) CeO_2_ exposure or at 18 h, 30 days or 60 days after a repeated (2 × 5 days) CeO_2_ exposure in control animals and for the low, mid and high exposure groups (*n* = 4 per group) in (**A**) mediastinal lymph nodes after a single exposure, (**B**) mediastinal lymph nodes after a repeated exposure, (**C**) liver after a single exposure (**D**) liver after a repeated exposure, (**E**) urine after a single exposure and (**F**) urine after a repeated exposure. Note that, in contrast to other organ tissues, cerium concentrations in lymph nodes are expressed in µg/g wet organ weight

Cerium concentrations have been determined in mediastinal lymph nodes, liver and urine for all dose groups following single and repeated exposures (Fig. 3).

Cerium concentrations in lymph nodes show a dose and time dependent increase compared to controls, indicating clearance from the lung over time (Fig. 3A and B, Additional file 2, Table S1 and S3). A small but significant increase in cerium concentrations compared to controls is found in livers in a dose and time dependent way, both after a single and repeated exposure (Fig. 3C and D, Additional file 2, Table S1 and S3 respectively). After a single 6-hour exposure, it takes between 18 h and 7 days post-exposure for translocation to occur from the lung to the liver (Fig. 3C). After 18 h post-exposure, there is only a single rat that shows an increase in cerium content in the liver (Fig. 3C). A dose dependent increase in cerium concentration is found in urine when collecting urine right after exposure (the first 18 h after exposure), but is no longer above the limit of detection at later timepoints (Fig. 3E and F).

**Fig. 4:**
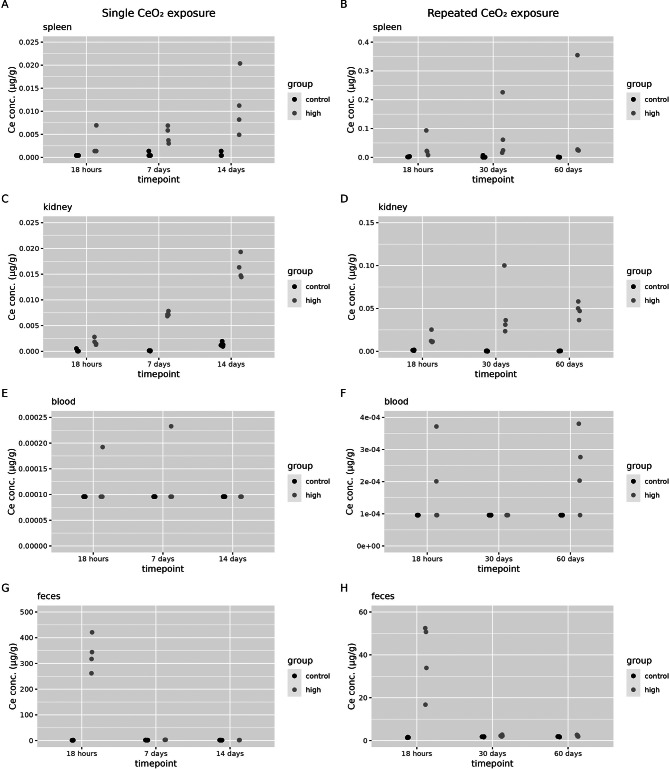
Biodistribution of cerium to secondary compartments. Cerium concentrations (in organ dry weight or in µg/g per excreta) were assessed 18 h, 7 days and 14 days after a single (1 day) CeO_2_ exposure or at 18 h, 30 days or 60 days after a repeated (2 × 5 days) CeO_2_ exposure in control animals and for the high exposure groups (*n* = 4 per group) in (**A**) spleen after a single exposure, (**B**) spleen after a repeated exposure, (**C**) kidney after a single exposure (**D**) kidney after a repeated exposure, (**E**) blood after a single exposure and (**F**) blood after a repeated exposure, (**G**) faeces after a single exposure and (**H**) faeces after repeated exposure

Cerium concentrations have been determined in the spleen (Fig. 4A and B), kidney (Fig. 4C and D), blood (Fig. 4E and F) and faeces (Fig. 4G and H) for the control group and high dose only following single and repeated exposures.

Cerium concentrations in spleen and kidney are significantly increased in the high exposure group compared to controls (see additional file 2, Table S1-S4 for statistical analysis). There is a time-dependent increase that is most clearly seen after a single exposure and a sampling time of 18 h, 7 and 14 days after exposure (Fig. 4A and C). There is almost no cerium detected in blood (Fig. 4E and F). After a single exposure, there is an occasional rat that has a slightly higher cerium concentration in blood compared to controls. After a repeated exposure, there is an increase in blood cerium content compared to controls in 3 out of 4 rats, 60 days after the exposure. The same trends are seen for the cerium tissue and excreta dose in microgram per tissue (Additional file 2, Figure S1 and S2).

**Fig. 5:**
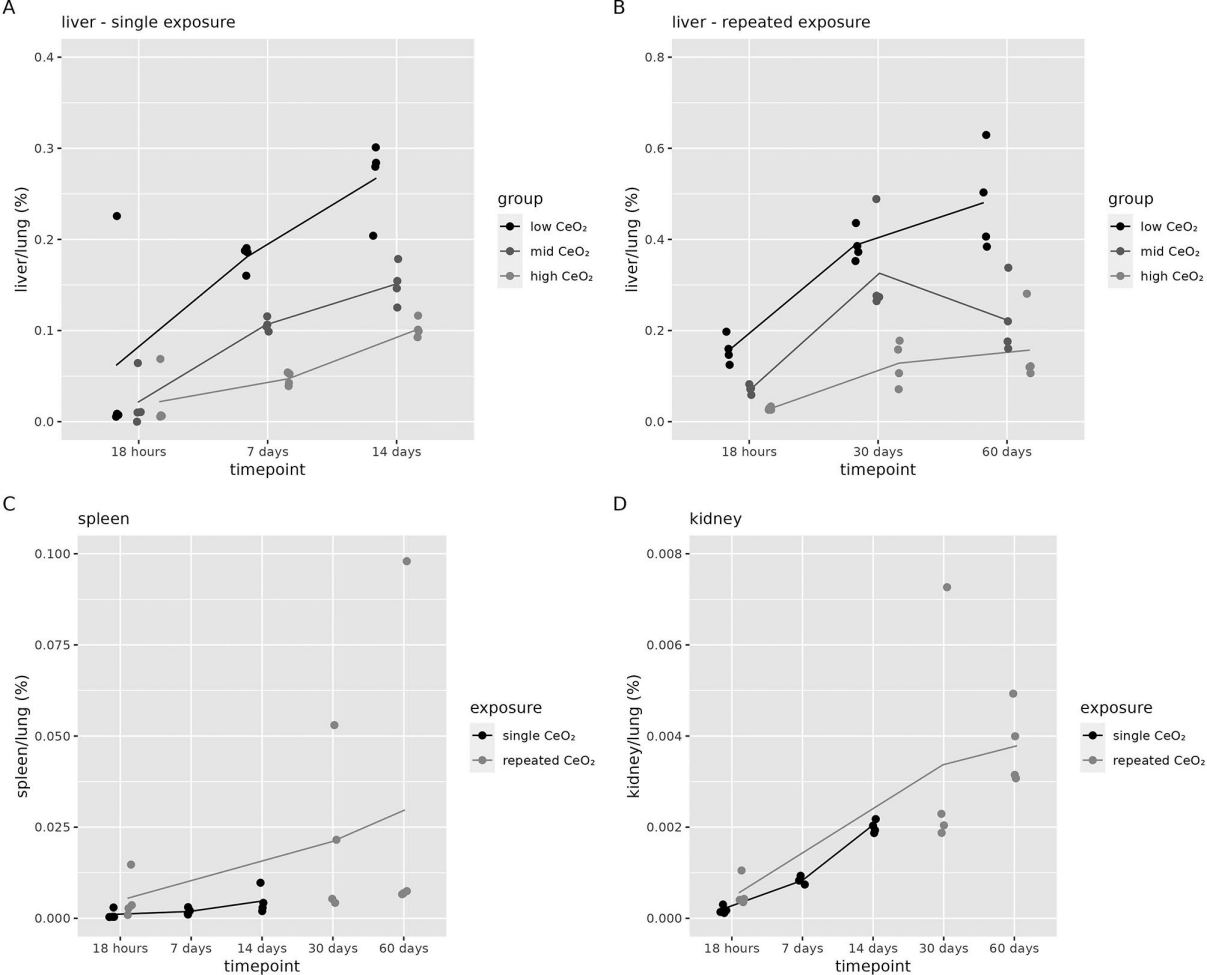
Secondary tissue content of cerium. Cerium secondary tissue content normalised to contemporaneous lung content and corrected for background levels measured in controls in (**A**) liver after a single exposure for the low, mid and high dose group (**B**) liver after repeated exposure for the low, mid and high dose group, (**C**) spleen for the high dose group only, after single and repeated exposure and (**D**) kidney for the high dose group only, after single and repeated exposure (*n* = 4 per group)

The relative translocation to secondary organs as a function of the contemporaneous lung content per applied dose group after a single and repeated exposure, is presented in Fig. 5. For this calculation only liver, spleen and kidney levels where the total amount of cerium was significantly different from the control group could be used (Additional file 2, Figure S1 and S2 for graphical representations and statistical analysis in Table S1 and S2).

The relative amount of cerium in the liver as a function of the contemporaneous lung content is around 0.5% or less, 60 days after a repeated exposure. This is higher compared to that in spleen (0.03%, 60 days after a repeated exposure) and kidney (0.004%, 60 days after a repeated exposure), but is still low. After a single exposure, there is a constant increase over time in relative liver over lung cerium content, whereas after repeated exposure for the mid and high, a maximum seems to be reached at 30 days after exposure. In spleen and kidney there is a constant increase in cerium content over time that does not seem to reach a plateau.

This analysis shows that the highest percentage of translocation of the initially deposited lung dose is to the liver (compared to the kidney and spleen) for the low dose group after repeated exposure. The translocation percentage is expected to be a little higher than 0.23% (for the low dose group the amount in the liver is 0.061 µg at t = 60 days divided by average lung dose of 26.3 µg corrected for the background at t = 18 h, see additional file 2, Figure S1), since we determined the lung burden at t = 18 h and not at 0 h after exposure.

**Fig. 6:**
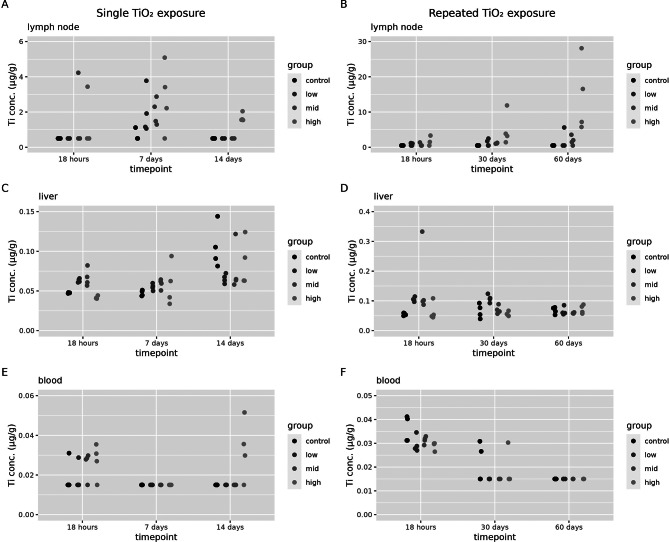
Biodistribution of titanium to secondary compartments. Organ concentrations (in µg/g organ dry weight) and blood concentrations (in µg/g) were assessed 18 h, 7 days and 14 days after a single (1 day) TiO_2_ exposure or at 18 h, 30 days or 60 days after a repeated (2 × 5 days) TiO_2_ exposure in control animals and low, mid and high exposure groups (*n* = 4 per group) in (**A**) mediastinal lymph nodes after a single exposure, (**B**) mediastinal lymph nodes after a repeated exposure, (**C**) liver after a single exposure (**D**) liver after a repeated exposure, (**E**) blood after a single exposure and (**F**) blood after a repeated exposure. Note that, in contrast to other organ tissues, titanium concentrations in lymph nodes are expressed in µg/g wet organ weight

Titanium concentrations in lymph nodes, liver and blood have been determined in all exposure groups (Fig. 6).

Titanium concentrations in lymph nodes are significantly increased over controls after a single exposure and repeated exposure in the high dose group (Fig. 6A and B, Additional file 3, Table S1 and S3, respectively). Especially after repeated exposure, clearance from the lung to the lymph nodes increases over time for the high dose group, but not for the low and mid dose (Fig. 6B). Unfortunately, background titanium levels in the liver are high in controls. Only after repeated exposure, when the results are expressed as titanium dose (not concentrations), there is a significant increase after repeated exposure (Additional file 3, Figure S1 and Table S3). After a single exposure, a dose dependent increase in titanium concentrations is found in blood 14 days after exposure (Fig. 3E, Additional file 3, Table S1).

**Fig. 7:**
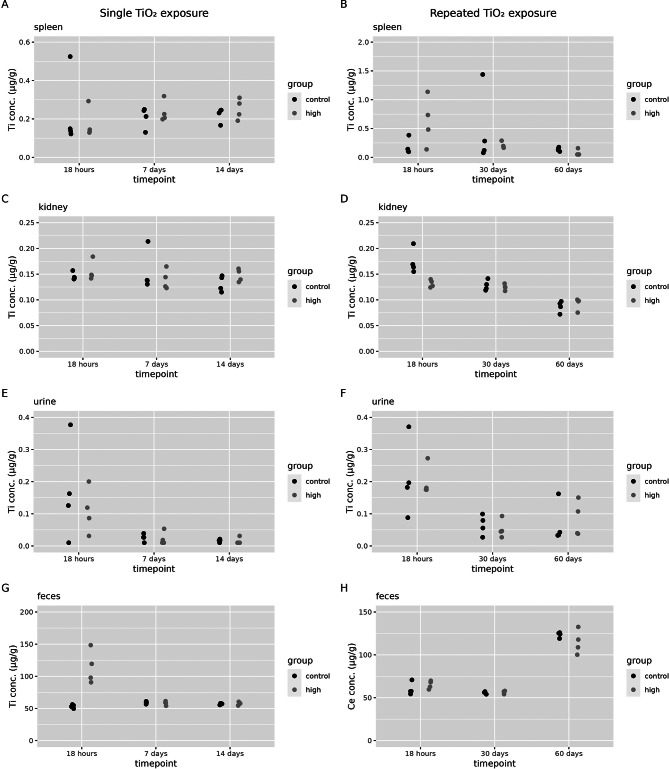
Biodistribution of titanium to secondary compartments. Titanium concentrations (in organ dry weight or in µg/g per excreta) were assessed 18 h, 7 days and 14 days after a single (1 day) TiO_2_ exposure. Or at 18 h, 30 days or 60 days after a repeated (2 × 5 days) TiO_2_ exposure in control animals and for high exposure groups (*n* = 4 per group) in (**A**) spleen after a single exposure, (**B**) spleen after a repeated exposure, (**C**) kidney after a single exposure (**D**) kidney after a repeated exposure, (**E**) urine after a single exposure and (**F**) urine after a repeated exposure, (**G**) faeces after a single exposure and (**H**) faeces after repeated exposure

Titanium concentrations in spleen, kidney, urine and faeces were determined in the controls and high exposure groups first (Fig. 7).

Since these organs/compartments did not show an increase in titanium content compared to controls levels, except in faeces 18 h after a single exposure to 20 mg/m^3^ TiO_2_, the low and mid dose groups were not further analysed. This increase in faeces concentration shortly after exposure is most likely linked to the initial mucociliary clearance of titanium from the bronchi and subsequent swallowing, and does not represent excretion after absorption.

**Fig. 8:**
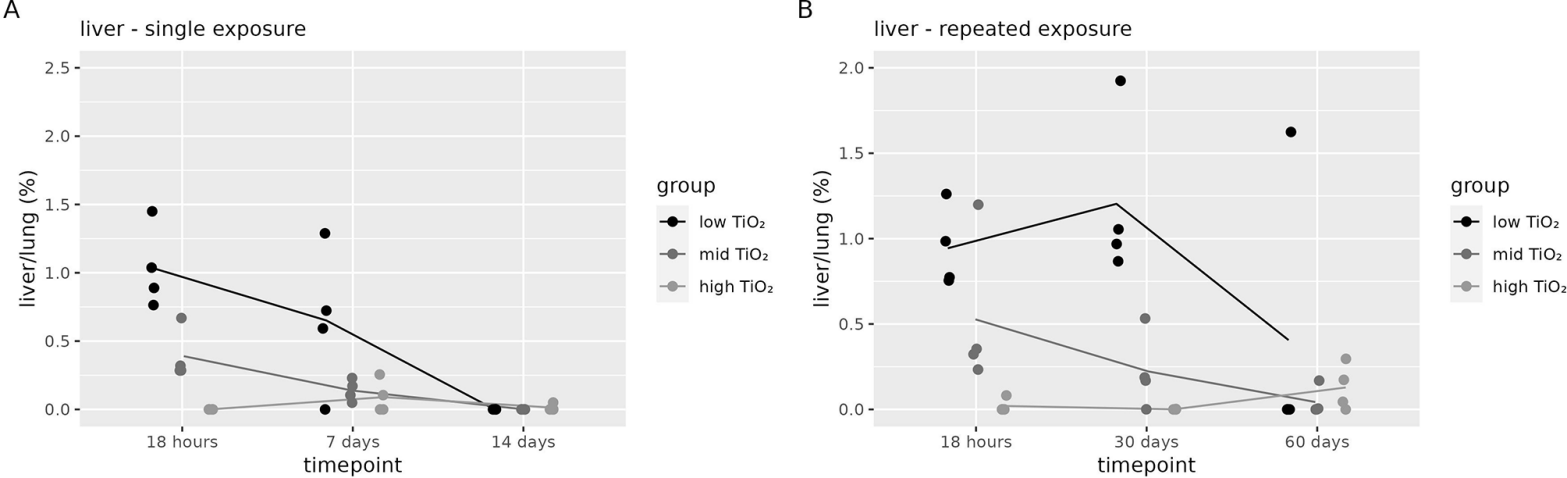
Secondary tissue content of titanium. Titanium content normalised to contemporaneous lung content in (**A**) liver after a single exposure for the low, mid and high dose group (**B**) liver after repeated exposure for the low, mid and high dose group after single and repeated exposure respectively (*n* = 4 per group)

The tissue dose in spleen and kidney after exposure to titanium were not significantly different from control groups (Additional file 3 for graphical representations in Figure S2 and statistical analysis in Table S1 – S4). Also, the liver tissue dose in the high dose group after a single exposure, and in the low dose after a repeated exposure were not significantly different from controls (Additional file 3, Table S1). Only the dose groups that were significantly different over controls could be used to calculate the relative amount of titanium in the liver over the contemporaneous lung content (Fig. 8).

The relative amount of titanium in the liver compared to the amount in the lung (around 1%, 30 days after a repeated exposure) is higher as compared to cerium (0.5% or less, 60 days after a repeated exposure). In the low dose group, the relative titanium increase in the liver is higher compared to the mid and high dose, which is also comparable to the observations with cerium. The relative liver to lung content after a repeated exposure over time seems to be similar or slightly increased for the low dose group, while after a single exposure as well as for the mid and high dose group there is a relatively fast decrease over time (Fig. 8B). The highest percentage of translocation of the initially deposited lung dose goes to the liver for the low dose group after repeated exposure. The translocation percentage is expected to be a little higher than 0.94% (i.e., the amount in the liver of 0.20 µg at t = 18 h corrected for background divided by average lung dose of 21.8 µg corrected for the background at t = 18 h for the low dose group, see additional file 3, Figure S1), since we determined the lung burden at t = 18 h and not at 0 h after exposure.

When comparing the relative liver to lung cerium content after a single exposure (Fig. 5A) to titanium (Fig. 8A), the relative amount of cerium is increasing while the relative amount of titanium is decreasing. After a repeated exposure, the relative cerium liver content (Fig. 5B) is still increasing in the low dose group or reaching a plateau for the mid and high dose group, while the relative titanium liver content is decreasing in the mid and high dose group (Fig. 8B).

### Toxicological assessment

No CeO_2_ or TiO_2_ exposure-related changes in body weight, organs weights (liver, heart, thymus, spleen, kidney, pancreas and brain) were found (raw data can be found in additional file 10 and 11).

**Fig. 9:**
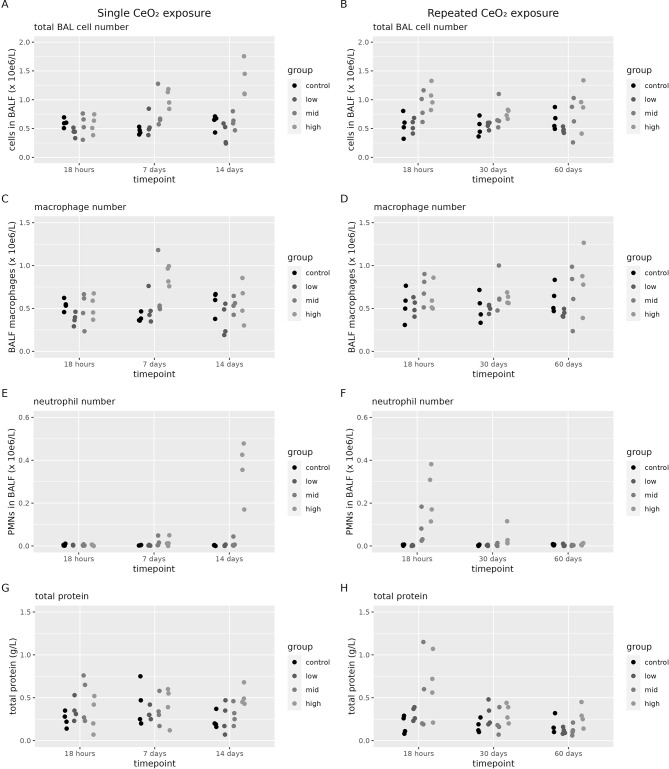
Lung toxicity after CeO_2_exposure. BALF analysis of toxicological markers assessed at 18 h, 7 days or 14 days after a single (1 day) CeO_2_ exposure. Or at 18 h, 30 days or 60 days after a repeated (2 × 5 days) CeO_2_ exposure (*n* = 4 per group) (**A**) Total cell count after a single exposure, (**B**) Total cell count after a repeated exposure, (**C**) total number of macrophages after a single exposure, (**D**) total number of macrophages after a repeated exposure, (**E**) total number of neutrophils after a single exposure, (**F**) total number of neutrophils after a repeated exposure, (**G**) total protein after a single exposure and (**H**) total number of protein after a repeated exposure

**Fig. 10:**
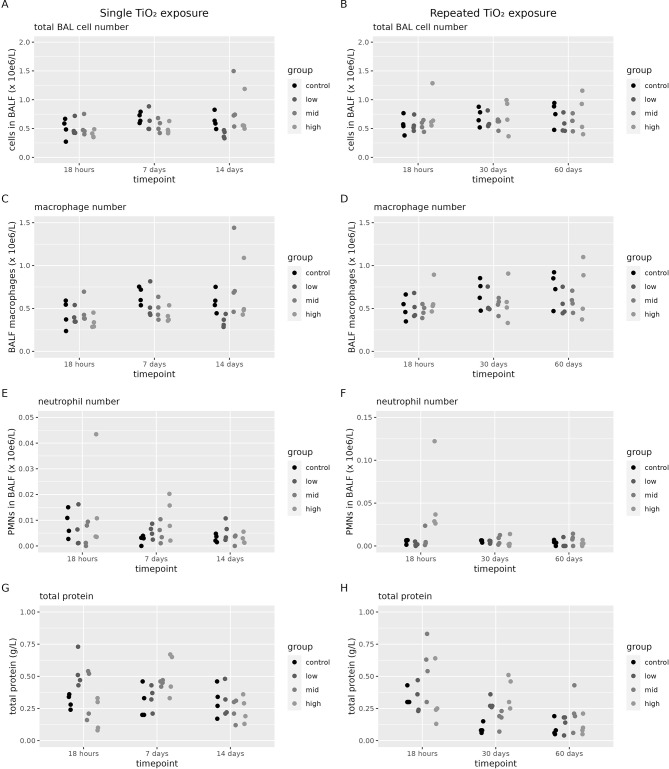
Lung toxicity after TiO_2_ exposure. BALF analysis of toxicological markers assessed at 18 h, 7 days or 14 days after a single (1 day) TiO_2_ exposure. Or at 18 h, 30 days or 60 days after a repeated (2 × 5 days) TiO_2_ exposure (*n* = 4 per group). (**A**) Total cell count after a single exposure, (**B**) Total cell count after a repeated exposure, (**C**) total number of macrophages after a single exposure, (**D**) total number of macrophages after a repeated exposure, (**E**) total number of neutrophils after a single exposure, (**F**) total number of neutrophils after a repeated exposure, (**G**) total protein after a single exposure and (**H**) total number of protein after a repeated exposure

BALF was assessed for total cell counts, differential cell counts, total protein and LDH content after single and repeated exposure to CeO_2_ (Fig. 9) and TiO_2_ (Fig. 10). There was no increase in LDH content above the detection limit (25 U/L) after exposure to either ENMs (data not shown).

After CeO_2_ ENM exposure, we observed a dose-dependent increase in total cell numbers, 7 days and 14 days after a single exposure (Fig. 9A) as well as 18 h after a repeated exposure (Fig. 9B). The increase in total cell numbers at day 7 after a single exposure can be attributed to an increase in the number of macrophages (Fig. 9C), while the increase at 14 days after a single exposure (Fig. 9E) and 18 h after a repeated exposure can be attributed to an increase in the number of neutrophils (Fig. 9F). For the high dose group, 30 days after repeated exposure the number of neutrophils remain significantly increased over controls (Fig. 9F). No statistically significant changes were found in total protein levels after a single exposure to CeO_2_ (Additional file 4, Table S1). A dose-dependent increase in total protein levels was found 18 h after a repeated exposure to CeO_2_ (Fig. 9H).

After TiO_2_ exposure, we observed a dose-dependent increase in neutrophil numbers, 18 h after a repeated exposure (Fig. 10F). No significant exposure-related changes compared to the controls were found in total cell numbers, number of macrophages and total protein (Additional file 4, Table S1).

To determine the dose for both CeO_2_ and TiO_2_ that induces lung inflammation and make a comparison on their relative potencies, the pulmonary responses as a function of the retained dose expressed as mass per timepoint were analyzed using the Benchmark dose approach. Table 4 summarizes the benchmark response (BMR) for the total cell number, number of macrophages, number of neutrophil granulocytes and total protein content in the BALF including the upper (BMDU) and lower (BMDL) confidence limits after exposure to CeO_2_. The underlying dose-response curves can be found in Figures S1-S8 of additional file 9.

**Table 4:** Dose-response analysis of toxicological markers after single and repeated exposure to CeO_2_

Endpoint	BMR	Exposure	Timepoint	BMDL (µg) *	BMDU (µg)*	BMDU/BMDL ratio (-)*	
Total cell number	100%	Single	18 h	330	Inf	Inf	
			7 days	84	260	3.1	
			14 days	59	190	3.2	
		Repeated	18 h	200	480	2.4	
			30 days	270	1200	4.4	
			60 days	160	620	3.9	
Fraction macrophages^#^	20%	Single	18 h	530	Inf	Inf	
			7 days	250	Inf	Inf	
			14 days	70	80	1.1	
		Repeated	18 h	120	130	1.1	
			30 days	310	340	1.1	
			60 days	420	770	1.8	
Fraction meutrophils	20%	Single	18 h	520	Inf	Inf	
			7 days	320	650	2.0	
			14 days	96	110	1.1	
		Repeated	18 h	160	Inf	Inf	
			30 days	420	510	1.2	
			60 days	720	Inf	Inf	
Total protein concentration	100%	Single	18 h	66	830	13	
			7 days	56	Inf	Inf	
			14 days	110	Inf	Inf	
		Repeated	18 h	130	890	6.8	
			30 days	120	680	5.7	
			60 days	110	690	6.3	

# Inverse macrophages

*rounded to two significant digits

Table 5 summarizes the outcome of the toxicological assessment after TiO_2_ exposure. The underlying dose-response curves can be found in Figures S9-S13 of additional file 9.

**Table 5:** Dose-response analysis of toxicological markers after single and repeated exposure to TiO_2_

Endpoint	BMR	Exposure	Timepoint	BMDL (µg) *	BMDU (µg)*	BMDU/BMDL ratio (-)*
Total cell number	100%	Single or repeated	All timepoints	No dose response		-
Fraction macrophages^#^	20%	Single or repeated	All timepoints	No dose response		-
Fraction neutrophils	20%	Single	18 h	180	380	2.1
			7 days	180	420	2.3
			14 days	730	inf	Inf
		Repeated	18 h	360	500	1.4
			30 days	1000	Inf	Inf
			60 days	210	Inf	Inf
Total protein concentration	100%	Single or repeated	All timepoints	No dose response		-

# Inverse macrophages

*rounded to two significant digits

CeO_2_ exposure is more potent in inducing an increase in total cell numbers, with an increased contribution of macrophages compared to TiO_2_. For example, a BMDL of 59, 70 and 56 µg is derived for the increases in the total cell count, the fraction of macrophages and total protein content in the BALF after a single exposure at timepoint t = 14 days, while no clear relation between dose and response was observed after exposure to TiO_2_, neither after a single or repeated exposure for all timepoints.

## Discussion

The biodistribution experiments described in the here presented study provided data, obtained under comparable experimental conditions, on distribution to organs or tissues for metal ENMs after inhalation that could serve as input for kinetic modelling (such as e.g. PBK model parametrisation). Organs include mediastinal lymph nodes, liver, spleen and kidney besides the lungs as well as providing measurements in urine and faeces to estimate absorption and elimination from the body. Mediastinal lymph nodes that are in close proximity to the lung receive an increased metal concentration upon increasing exposure to CeO_2_ NM-212 and TiO_2_ NM-105. This has been previously described for CeO_2_ NM-212 and a TiO_2_ ENM with similar primary particle size, BET surface area and rutile/anatase proportions as used here [13, 16, 21]. Uptake by phagocytic cells in the liver and spleen (the reticuloendothelial system) is the most common pathway for the body to remove ENMs with sizes above 6 nm from the blood and this is a relatively fast process [23, 24]. This also explains why there is no cerium and hardly any titanium detected in blood, except in the high dose group shortly (within 18 h) after a single exposure. Surprisingly, in 3 out of 4 animals, increased titanium concentrations above control levels were also observed in blood 14 days after a single exposure, while titanium is not detectable 7 days after a single exposure. This phenomenon may be the result of delayed clearance, but the high background and more variation in Ti measurements hamper a definite conclusion.

It should be noted that the limit of detection (LOD) by ICP-MS differs between titanium and cerium. The LOD for titanium in this study was at least 200 times higher compared to that for cerium. This can be attributed to several reasons including spectral interferences and higher background concentrations for titanium. The latter could partly be due to higher titanium concentration in the feed of the rat compared to cerium (Additional file 7, table S7). This is reflected in more variation in the titanium concentration measurements in secondary organs and excreta and more difficulty in detecting titanium concentrations above control levels.

By choosing two metal oxide nanomaterials, CeO_2_ NM-212 and TiO_2_ NM-105, that are both considered to be poorly soluble in biological fluids and with similar primary particle size characteristics (around 20–30 nm) and, as it turned out, also have similar aerodynamic aerosol characteristics (MMAD of around 1 μm) in our aerosol generation set-up, and using the same exposure durations for the single and repeated exposures as well as timepoints in the post-exposure period, we were able to generate comparable doses in the lung and this allows studying the influence of other variables on the biokinetics of ENMs.

## Biodistribution determined around and below toxicological effects

The retained lung burden (at t = 18 h) after a repeated exposure to an aerosol concentration of ~ 5 mg/m^3^ of CeO_2_ and TiO_2_ ENMs, resulted in a two-fold higher retained total lung dose compared to a single exposure to a four times higher concentration (~ 20 mg/m^3^) of CeO_2_ and TiO_2_ (Table 3). Note that the rapid first phase of tracheobronchial clearance via the mucociliary escalator has already taken place within the first 18 h [25]. The applied ‘concentration times exposure duration’ (Cxt) protocol resulted in a linear increase in lung burden with increasing exposure time of the rats in the nose-only tubes for the low, mid and high exposures for both ENMs in the same range of 10 to 350 µg per lung.

Analysis of differences in pulmonary toxicity potency of the two ENMs showed that a BMDL of 50–70 µg of cerium after a single exposure, leads to a statistically significant increase in the total cell number, the fraction of macrophages and total protein content in the BAL fluid (BALF), while a slightly higher benchmark dose of cerium is needed to induce a neutrophil influx (BMDL of 96 µg at t = 14 days). This retained dose is reached in the highest dose group after a single exposure (145 ± 12 µg, Table 3). At a retained lung dose of 91 ± 11 µg (Table 3) no significant pulmonary effects were induced by titanium. Even when converting the dose to surface area instead of mass, TiO_2_ ENM is less potent in inducing pulmonary inflammation compared to CeO_2_ ENM. The BET specific surface area of the TiO_2_ ENM has been reported as twice as high compared to the CeO_2_ ENM (51 m^2^/g compared to 27 m^2^/g, respectively.

After repeated exposure to TiO_2_ ENM, the number of neutrophils increase 18 h after exposure in the mid and high dose group (Fig. 10F). This inflammatory response did not persist over the prolonged post-exposure time of 30–60 days in the highest dose group. Whereas we observed an increased neutrophil response 18 h as well as 30 days after repeated exposure to CeO_2_ for the high dose group only (Fig. 9F). Persistence of the inflammatory response has been observed in other studies for CeO_2_ ENMs [11, 15]. No effect on LDH release was found at any of the dose levels in the present study, which is in line with previously reported ‘lowest-observed-adverse-exposure levels (LOAELs) over ~ 400 µg for CeO_2_ NM-212 [11, 14–16] and TiO_2_ NM-105 or P25 exposures [14, 19] based on cellular damage markers such as LDH and BAL cellularity. The biodistribution pattern to secondary organs for cerium has most likely not been compromised by barrier damage based on only a slight increase in total protein content in the BALF after repeated exposures and no effect on LDH release. For the low dose CeO_2_ group, no effects on pulmonary markers are seen. The biodistribution to secondary organs may theoretically have been influenced at the high and probably also mid dose levels by an inflammatory response and subsequent prolonged increased permeable blood-lung barrier judging from the increased BAL cellularity (macrophage and neutrophils numbers). We do see that when expressing the cerium liver tissue content normalised to the contemporaneous lung content, 30 days after repeated exposure, a plateau seems to be reached in the mid and high dose group, but not for the low dose group. This is not observed for the kidney and spleen content, nor after a single exposure. It remains speculative that reaching such a plateau in the liver has a connection to the pulmonary effects in the lung. It could also be related to a certain absolute dose above which conditions are created that influence the elimination rate of cerium from the liver or it could be related to differences in bioprocessing of cerium. It has been shown that there are difference in bioprocessing in the liver com[pared to the spleen after iv infusion [26]. From a perspective of risk assessment and the design of future toxicokinetic studies of poorly soluble particles, we consider it important to include at least 1 higher concentration that does lead to pulmonary effects [27]. To better understand the biodistribution behaviour of poorly soluble ENMs in general, exposures leading to low (no pulmonary effects), mid (onset of pulmonary effects) and high doses (leading to irreversible pulmonary effects) in the lung are all relevant, since we do not fully understand the consequences yet for biodistribution to secondary organs.

After TiO_2_ exposure, only the applied high dose group after repeated exposure shows transient effects on neutrophilic increase at the earlier timepoints. No effects on macrophage recruitment, total protein or LDH content in the BALF are found. Therefore, we expect that the limited pulmonary toxicity of the retained titanium lung doses did not play a role in the TiO_2_ biodistribution to secondary organs.

### Metal concentrations in different lung compartments

The relative highest concentration of cerium and titanium is found in the lavaged lung tissue. This is most likely representative of poorly soluble CeO_2_ and TiO_2_ ENMs present inside the lung tissue or engaged with the tissue surface. This includes ENMs inside a small portion of the phagocytic cells such alveolar macrophages that are not washed from the lung during the lavage. Previous microscopic studies have also shown that 20 nm sized TiO_2_ nanoparticles were found in alveolar epithelial type-1 and type-2 cells and much smaller numbers in the vascular endothelia [28–30]. Different types of TiO_2_ ENMs were shown to be quarantined on the surface of the alveolar cell wall and cycle between uptake in macrophages, release by macrophages and re-uptake of epithelial cells [31]. Earlier work by Motskin et al. showed that macrophages, but also A549 epithelial cells in vitro, can sequester a high amount of hydroxyapatite nanoparticles in a specific surface cell compartment that is still connected to the extracellular space [32]. The second highest concentration of cerium and titanium is found in the (phagocytic) cells obtained by BAL and the lowest concentration is present in lavage fluid, representing the portion that is freely available on the alveolar surface. A similar relative division over the lung compartments has been found for radioactively labelled ^141^CeO_2_ nanoparticles 24 h after a single intratracheal instillation [24] or after inhalation of radioactively labelled [^48^V]TiO_2_ nanoparticles [17].

### Lung clearance

The increase in the estimated half-time from 58 up to 134 days after the repeated exposure to CeO_2_ is indicative of impaired clearance. This result aligns with the strong neutrophil response 14 days after a single exposure as well as the persistent neutrophilic response 30 days after repeated exposure for the high exposure group. Previous studies showed that either the elimination half-time for CeO_2_ from the lung could not be established since the post-exposure period was too short [16], or that the elimination half-time was estimated as 67, 69, 108 and 224 days after 90 days exposure to 0.1, 0.3, 1.0 and 3.0 mg/m^3^ CeO_2_ NM-212 [15] and was estimated to be 86, 114, 164 and 200 days after 2 years exposure to 0.1, 0.3, 1.0 and 3.0 mg/m^3^ CeO_2_ NM-212 respectively [13]. After TiO_2_ exposure, there is no indication of impaired clearance. The estimated half-time stays within the same range of 18–31 days. Previously reported lung elimination half-times following the inhalation of low concentrations of CeO_2_ and TiO_2_ were typically in the range of 40–60 days [12, 20] and 25 days for primary nanosized spark generated TiO_2_ NPs [17].

### Biotransformation

The method of detection used here unfortunately does not allow to discriminate between particles or ions present in the different organs and excreta. The increased cerium levels in urine 18 h after exposure could be caused by very small particulates that are already present in the NM-212 ENM sample. The nominal primary particle size is reported to be 28.4 ± 10.4 nm (mean Ferets diameter based on scanning transmission electron microscopy images) and is larger than the corresponding crystallite size by X-ray diffraction analysis. This is not surprising as a particle may consist of several different crystallites. TEM images show irregular and non-homogeneous primary particle size variation, but this size of the smallest particles are not recorded [9]. In order to pass the glomerular filtration barrier in the kidney, nanoparticles need to be smaller 6–8 nm to be excreted by the kidney [23].

A less likely explanation of the urine results is that particles are reduced in size due to biotransformation processes. Hydro-thermically derived CeO_2_ NPs were shown to undergo different in vivo bioprocessing in the liver and spleen over time [26]. Bioprocessing has been defined as the “dynamic chemical and/or physical breakdown of nanoparticles at the cellular and subcellular level, a process that can be followed by in vivo formation of new reaction products including ions, nuclei and growth of second generation nanoparticles ” [33]. But since a reduction in particle size was found 90 days after in vivo processing of CeO_2_ nanoparticles in the liver [34], it is unlikely that the rapid increase in urine excretion that is observed here is due to a reduction in size by bioprocessing. The formation of smaller particles containing cerium could, however, result in a higher translocation following pulmonary exposure compared to titanium dioxide (TiO_2_). After a single instillation in the lung of mice, pulmonary deposited CeO_2_ or TiO_2_ ENMs translocated to the liver with similar translocation rates. A reduction in the particle size distribution of CeO_2_ in the lung and the liver indicated bio-transformation. A similar analysis was not possible for TiO_2_ ENMs due to the relatively high size Limit of Detection of 50 ± 60 nm by single particle ICP-MS for this type of ENM [35].

### Recommendations for future research

Based on the work described here, there are a number of considerations that could provide guidance for future studies advancing PBK models for poorly soluble ENMs. Based on the lung elimination half-time and the fact that this could not be accurately determined after a single CeO_2_ exposure and a follow-up period of 14 days, we would recommend to prolong the post-exposure beyond 14 days to for example 60 days, especially when reduced clearance is expected. In other work where we used this dataset [36], we showed that reducing the number of animals per time point does not lead to a significant change in the estimated elimination rate. In future research, it would be interesting to investigate the trade-off between decreasing the number of animals per time point and increasing the number of time points when designing an in vivo biokinetics study.

## Conclusions

We have generated high quality datasets for CeO_2_ (28.4 ± 10.4 nm) and TiO_2_ (21.6 ± 1.5 nm) ENMs. The study design allows comparison of two poorly soluble ENMs with similar primary and aerosol agglomerated size and indicated several differences in the lung clearance and biodistribution pattern. The relative distribution of cerium and titanium over the different lung compartments is similar, with the highest concentration in the lavaged lung tissue and the lowest concentration in the BAL fluid. Comparable deposited lung doses did lead to increased lung elimination half-times for cerium but not for titanium. Cerium is also more potent in inducing persistent inflammation compared to titanium even when converting the dose to surface area instead of mass. Cerium is detected in more secondary organs (lymph nodes, liver, spleen and kidney) compared to titanium (lymph nodes and liver), while the relative amount of titanium in the liver compared to the contemporaneous lung dose is higher compared to cerium. Toxicological investigation indicated that the biodistribution has been studied here under conditions where at least two out of three applied dose regimes did not lead to pulmonary inflammation or pulmonary damage.

### Methods

#### Animals

Young male Wistar outbred rats (HsdCpb: WU) were obtained from a colony maintained under specific pathogen-free (SPF) conditions by Envigo, Netherlands with body weights of 240 +/- 20 g. To reduce the total number of animals and costs, we have used a single sex. We have chosen male rats as they have a higher inhalation rate leading to higher deposited doses in the lung [37], increasing the chance of detection of the metal composition of the ENMs by ICP-MS. The animals are housed for acclimatization for one and a half weeks. The animals were around 10 weeks of age at the start of the experiment.

Rats were trained to get accustomed to the nose-only tubes (IET-200 EMMS, UK). On the first day the rats remained inside the tube for 30 min, on the second day for 1 h and on the third day for 2 h. Control and treated animals were exposed for a maximum duration of 6 h. A single aerosol concentration was generated for the different exposure groups. A dose-equivalent was derived by multiplying the duration of exposure with the exposure concentration (Cxt) as described previously [16]. This study design choice has the additional advantage of keeping the agglomeration status of the ENM and the MMAD the same while obtaining different dose levels in the lung. Animals that received a low or mid dose of nanomaterial (by inhaling the test atmosphere for 30 min– or 2 h), remained in the inhalation tube for the full 6 h. When not exposed to nanoparticles, these animals were transferred/attached to the clean air nose-only exposure chamber.

First, a pilot study for TiO_2_ and CeO_2_ was performed (*n* = 2 per group) to assess whether cerium and titanium concentrations could be detected in the tissue by ICP-MS and to check whether the exposure would lead to cell damage or an inflammatory response in the lung. In two separate pilot studies, rats were exposed to a single nose-only inhalation for 6 h to (a) clean air and to an aerosol with a target concentration of 20 mg/m^3^ TiO_2_ NM-105, and (b) clean air and CeO_2_ NM-212 (*n* = 2). After 18 h, animals were sacrificed by bleeding via the abdominal aorta and lungs were flushed to obtain the BALF. Blood, BALF, lung and liver tissue as well as the mediastinal lymph nodes were collected. BALF was analysed for cell damage and inflammatory markers. Lavaged lung tissue, BAL fluid, BAL cell pellet and liver were analysed for titanium or cerium content. The results from the pilot study confirmed the detection of titanium and cerium in lungs, lymph nodes and liver, and confirmed that the target concentration and at least one of the exposure durations would not lead to cellular damage or pulmonary inflammation (Additional file 1).

The pilots were followed by the main studies: a single exposure to a target concentration of 20 mg/m^3^ CeO_2_ or TiO_2_ for 30 min, 2 or 6 h, and a repeated exposure to a target concentration of 5 mg/m^3^ CeO_2_ or TiO_2_ during 30 min, 2 h or 6 h for 5 consecutive days, 2 days rest with no exposure followed by 5 consecutive days of exposure. Following the single exposure, groups of 4 rats were sacrificed 18 h, 7 days or 14 days post exposure. Following repeated exposure, groups of 4 rats were sacrificed 18 h, 30 days or 60 days after the final exposure. Lungs were flushed similarly as described for the pilot study. Blood, BALF, mediastinal lymph nodes, lung, liver, spleen, thymus, pancreas, heart, brain and kidney tissue were collected. Urine and excreta were collected in metabolic cages. Rats were housed individually without cage enrichment. Cage enrichment could potentially soak up excretions and reduce the accuracy of the collection. The room temperature was increased up to 2 degrees to accommodate potential heat loss from the body due to the individual housing. Food (Teklad global 2018 S, Envigo) and drinking water was provided ad libitum, with the exception of the time the animals remained inside the nose-only tube. Feed pellets were checked for the total cerium and titanium content by ICP-MS (Additional file 2).

### Nanomaterials

Both CeO_2_ and TiO_2_ materials were aerosolized from a dry powder. The same pristine CeO_2_ NM-212 nanomaterial (CAS-nr 1306-38-3) was used as for the inhalation studies in the OECD Sponsorship program that has been published previously [11, 16]. The nanomaterial has been stored in dark bottles under argon or nitrogen. The material has been extensively characterized in the OECD Sponsorship program [9] and has a primary particle size of 28.4 nm ± 10.4 (mean Ferets diameter from SEM pictures). The particles are crystalline with a material density of 7.65 g/cm^3^. It was estimated that the effective density is between 1.5 and 2.4 g/cm^3^ [38]. BET specific surface area is 27 m^2^/g [39].

Pristine TiO_2_ NM-105 (CAS nr: 13463-67-7) has been extensively characterized in the OECD Sponsorship program (Rasmussen et al. JRC report: Titanium Dioxide, NM-100, NM-101, NM-102, NM-103, NM-104, NM-105: Characterisation and Physico-Chemical Properties). TiO_2_ NM-105 is a photocatalytic type similar to P25 consisting of 25% rutile and 75% anatase crystal structure. XRD analysis confirmed the crystal structure for TiO_2_ NM-105 as anatase 86.3 ± 0.2: Rutile 13.7 ± 0.1 as previously reported (see Additional file 5, Figure S1) [10]. Primary particle size is 22.6 nm ± 1.4–21.6 nm ± 1.5 Feret mean +/- SD measured by 2 different labs), effective density of 4.3 g/cm^3^ (based on Teleki et al. (2008) and described in JRC report by Rasmussen) and BET surface area of 46 m^2^/g or 51 m^2^/g [39].

### Aerosol generation

The aerosol was generated from the CeO_2_ and TiO_2_ dry powder using a turntable dust feeder [40] and an eductor (Fox Valve Development Corp., Dover, NJ, USA; [41] supplied with humidified compressed air at 1.4 kg/cm^2^ (CeO_2_) or 1.2 kg/cm^2^ (TiO_2_). The total air-flow was at least 29.0 L per minute. A cyclone was used to remove large particle agglomerates (URG-2000-30EH). During the exposure, the relative humidity, air temperature and CO_2_ concentration were measured. A drawing of the test atmosphere generation set up is given in Additional file 10, Figure 6).

Particle number counts and size distributions were measured daily during the experimental period with a condensation particle counter, a scanning mobility particle sizer (SMPS, model 3936, TSI, consisting of differential mobility analyzer (model 3080) with a long DMA (model 3081) and condensation particle counter (model 3022 TSI) and an optical particle sizer (OPS, model 3330, TSI). The gravimetric aerosol mass concentration was measured hourly using a vacuum pump connected to a mass flow controller (Bronkhorst) on 47 mm Teflon filters (Teflo R2JP047, Pall) connected to one of the ports of the nose only chamber. The MMAD was determined using a Micro-Orifice Impactor (MOImodel 110). During the exposures a tapered element oscillating microbalance (TEOM, model 1400 A, Ruprecht&Pataschnick) was used to measure the fluctuations in the mass concentration.

### BALF

Animals were weighed before dissection under 75 mg/kg body weight ketamine/0.25 mg/kg body weight dexmedetomidine anesthesia. The abdomen and thorax were opened and blood was withdrawn from the abdominal aorta with a syringe. A cannula was placed in the trachea and the diaphragm was opened. The lungs were flushed once using 26.7 mL/kg body weight of saline. The flush consisted of 3 up and down movements. Only BAL data with a recovery > 60% was included in the final data analysis.

### ICP-MS analysis

ICP-MS was deemed to give enough detailed information on organ and excreta concentrations that could be converted to an internal tissue dose given the organ dry weights. However, this method of choice ruled out the possibility for a complete mass balance as, to our knowledge, there is no established method to accurately assess the metal content in the remaining carcass. Cerium and titanium levels were measured in a tiered approach. In case the high exposure group showed an increase compared to controls, the mid and low dose were measured as well.

For cerium sample preparation and detection, different weights of rat samples (between 20 mg and 550 mg) were weighted into the 18 mL quartz vials and various amounts of concentrated nitric acid (67–69% HNO_3_, PlasmaPure, SCP Science, Quebec, Canada) and/or ultrapure water (18.2 mΩ·cm at 21.5 °C, from a Millipore Element apparatus, Millipore, Milford, MA, USA) were added. The samples were digested in a microwave reaction system Multiwave 7000 (Anton Paar GmbH, Graz, Austria), using the following digestion program: ramp to T = 250 °C for 20 min, hold at T = 250 °C for 10 min, cool down for 30 min, starting pressure of 40 bar and max pressure of around 120 bar. After digestion, the samples were transferred to 15 or 50 mL disposable polypropylene tubes (Sarstedt AG & Co. KG, Germany) and diluted with UPW to a final sample weight of 10–40 g. Prior to ICP-MS analysis, the digested samples were further diluted 1.56- to 200-times with UPW or 2% HNO_3_ (see additional file 7, Table S1 for details on sample preparation procedure for each sample type).

For quality assurance, blank samples, laboratory duplicates and spiked samples were included in all analyses. Ultrapure water that was treated in the same way as the rat samples during the animal experiments was used as a blank sample. Blank samples were digested in the same way as the corresponding rat samples. At least three blank samples were prepared for every sample type. As no suitable biological reference material for CeO_2_ was available, rat samples from control experiments were spiked with different concentrations of ionic cerium (Ce) standards and digested in the same way as the rat samples. Between one and five spiked samples were prepared for every sample type. Information on the preparation of spiked samples is summarized in additional file 7, Table S2. All samples were weighed on an analytical Sartorius GENIUS ME balance (Göttingen, Germany).

The total mass concentration of cerium (Ce) in the digested samples was determined by inductively coupled plasma-mass spectrometry (ICP-MS) using an Agilent 8900 Triple Quadrupole ICP-MS instrument (Agilent Technologies, California, USA), equipped with a Micro Mist borosilicate glass concentric nebulizer, a Scott type double-pass water-cooled spray chamber, platinum cones and an auto sampler (SPS4, Agilent Technologies). The analysis was performed in single quadrupole mode without the use of any collision or reaction cell gas. Optimization of instrumental parameters (additional file 7, Table S3) was performed on daily basis for best sensitivity of Ce using an ionic standard solution that contained 1 µg/l of Ce in 2% HNO_3_ (prepared from the certified standard stock solution containing 1000 mg/L of Ce (PlasmaCAL, SCP Science, Baie D’Urfé, QC, Canada)).

Determination of the Ce mass concentrations was performed based on external calibration by measuring Ce standards in the concentration range of 0.002–50 µg/L with online internal standardization (10 µg/L solution of rhodium (Rh)) (Instrumental settings are reported in additional file 7, Table S3). Calibration and internal standards were prepared from standard solutions that contained 1000 mg/l of Ce or Rh, respectively (both provided by Plasma CAL, SCP Science). All standards were matrix-matched with the diluted samples (i.e. prepared in 2% HNO_3_). To reduce carry-over from previous measurement, extensive rinsing between the samples was performed with 4% HNO_3_ for 1 min, followed by rinsing with 2% HNO_3_ for 0.5 min.

The limit of detection (LOD) and limit of quantification (LOQ), calculated as 3- and 10-times standard deviation of the blank samples (*N* ≥ 3), respectively, are presented in additional file 7, Table S4. The LOD and LOQ values in samples varied for the different sample types due to different sample intake in the digestion step and the sample dilution prior to analysis (see additional file 7, Table S4). The average mass concentration of Ce determined in the blank samples was subtracted from the Ce mass concentrations determined in the corresponding rat samples.

For titanium sample preparation and analysis, depending on the amount of sample available, an analytical sample of 0.1 to 1 g was collected from each grinded and homogenized sample and brought into a perfluoroalkoxy (PFA) microwave digestion tube to which 6 mL of nitric acid (70% HNO_3_) and 2 mL of hydrofluoric acid (40% HF), were added. All subsamples were digested for 55 min in a MARS microwave system (CEM Corporation, Matthews, NC, USA). The temperature program was as follows: at 1600 W power from 20 to 120 °C in 15 min, then to 160 °C in 10 min, and then to 210 °C in 30 min and hold for 1 min. Following digestion and cooling to room temperature, ultra-pure water was added to a total volume of 50 mL. The extracts were shaken manually and diluted 2 times further.

The diluted digests were analyzed with a Thermo Finnigan Element 2 (Thermo Fisher Scientific GmbH, Bremen, Germany) sector-field high resolution inductively coupled plasma mass spectrometer (ICP-HRMS) to determine total-titanium (Ti). The Thermo Finnigan Element 2 was operated at a forward power of 1300 W and the argon gas flows were at the following settings; plasma, 15.4 L/min; nebulizer around 1 L/min and optimized daily; auxiliary, 1.2 L/min. The sample flow rate to the nebulizer was set at 0.5 mL/min. The ICP-HRMS was operated in medium resolution mode with Ti measured at m/z 46.95 to avoid interferences from ^36^Ar^12^C, ^32^S^16^O, and ^48^Ca. Interferences on m/z 46.95, e.g. ^31^P^16^O, ^14^N^16^O_2_^1^H and ^15^N^16^O_2_, are easily separated from Ti in medium resolution mode.

Quantification was based on Ti standards diluted in the same acidic matrix as the samples. The method was validated over a period of several days [42]. The repeatability and recovery of the total-Ti method was determined by spiking blank samples at 0.1 mg/kg with NM-104 TiO_2_ nanomaterial. The repeatability is < 15% and the recovery > 90%. The LOD of the method is depending on the sample intake (Additional file 7, Table S6). During sample analysis blank samples spiked with NM-104 and ionic Ti were analyzed as control samples with each series of samples. The recovery of total-Ti in NM-104 spiked samples ranged from 77 to 102% with an average recovery of 86% and a standard deviation of 8%. The recovery of total-Ti in ionic Ti spiked samples ranged from 84 to 119% with an average recovery of 99% and a standard deviation of 8%. Method blanks were determined by performing the complete procedure, however, without the addition of a sample. The total-Ti concentrations in the blanks were below the LOD.

### Lung elimination half-times

For both CeO_2_ NM-212 and TiO_2_ NM-105 and each exposure group separately, a linear regression model of the log of the total lung load against the post-exposure time (in days) was fitted (additional file 8 for more detailed description. The R function lm() from the R stats package was used (*R Core Team (2020). R: A language and environment for statistical computing. R Foundation for Statistical Computing, Vienna, Austria. URL*https://www.R-project.org/).

#### Statistics

The raw data files with the results of the elemental analysis as well as the toxicological assessment of CeO_2_ and TiO_2_ ENMs are available in additional file 10 and 11, respectively. BALF cytology and total protein data were log transformed and analyzed by two-way ANOVA. Cerium concentrations and/or total content (dose) in lavaged lung tisssue, BAL cell, BAL fluid, total lung, mediastinal lymph nodes, liver, spleen, kidney and urine (control, low, mid and high dose) were log transformed and analyzed by two-way ANOVA. The same statistical analysis was used to assess the titanium concentrations and/or content in lavaged lung tisssue, BAL cell, BAL fluid, total lung, mediastinal lymph nodes, liver and blood. A Bonferroni multiple comparison test was applied for those organs/excretions with control versus high dose group only (kidney, spleen, urine and faeces).

Benchmark dose–response analysis was performed on the retained lung dose versus several toxicological parameters using PROAST (version 70.2 https://www.rivm.nl/en/proast). Dose–response models were fitted to the data, a BMR was defined, and the lower and upper 95%-confidence limits (one sided, BMDL and BMDU, respectively) of the associated benchmark doses were derived from the fitted model as previously described [43]. A BMR of 100% change in response was chosen for the total number of BAL cells, total protein and LDH [11]. For effects on the fraction of differential cell types, such as macrophage and neutrophils, the BMR was set to 20% extra risk [43].

### Electronic supplementary material

Below is the link to the electronic supplementary material.

Supplementary Material 1

Supplementary Material 2

Supplementary Material 3

Supplementary Material 4

Supplementary Material 5

Supplementary Material 6

Supplementary Material 7

Supplementary Material 8

Supplementary Material 9

Supplementary Material 10

Supplementary Material 11

## Data Availability

Raw data on elemental analysis in biological fluids and organs, as well as on the toxicological parameters are provided in additional file 10 (for CeO_2_ ENM) and in additional file 11 (for TiO_2_ ENM).

## Abbreviations

ANOVA: Analysis of variance
BAL: Bronchoalveolar lavage
BMDL: Benchmark dose-response lower confidence limit
BMDU: Benchmark dose-response upper confidence limit
BMR: Benchmark dose-response
Ce: Cerium
CeO_2_: Cerium dioxide
CMD: Count median diameter, ENMs, Engineered Nanomaterials
gsd: Geometric standard deviation
ICP-MS: Inductively coupled plasma-mass spectrometry
LDH: Lactate dehydrogenase
LOD: Level of detection
LOQ: Limit of Quantitation
MMAD: Mass median aerodynamic diameter
PBK: Physiologically-based kinetic
sd: Standard deviation
TEM: Transmission electron microscopy
Ti: Titanium
TiO_2_: Titanium dioxide
UPW: Ultra pure water
