# Supplementary material for: Biodistribution of cerium dioxide and titanium dioxide nanomaterials in rats after single and repeated inhalation exposures
Source: Part Fibre Toxicol. 2024 Aug 14;21:33. doi: 10.1186/s12989-024-00588-4 (PMC11323389; doi:10.1186/s12989-024-00588-4)
Supplement: Supplementary file 1 — Supplementary Material 1 [file 12989_2024_588_MOESM1_ESM.docx]

**Additional file 1**

**Pilot studies**

To set the exposure concentration in the pilot study, multiple-path particle dosimetry (MPPD V3.14) modelling was used to estimate the deposited fractions in the different lung regions. The mass median aerodynamic diameter (MMAD) was assumed around 1.4 µm (GSD 1.0) for both ENMs. The chosen species/geometry was Long Evans asymmetric on stomach nose-only exposure, FRC 4 ml, URT volume 0.42ml, density 7.65 g/cm^3^, deposition only. The results were head (0.4766), tracheobronchial (0.0345) and pulmonary deposition (0.0828). Total deposition was estimated to be 0.5958 Based on this result, we decided to keep the mass concentration for the pilot studies on 20 mg/m^3^.

In the pilot exposure studies for TiO_2_ and CeO_2_, ICP-MS was used to detect titanium (Ti) and cerium (Ce).

In Table S1, the aerosol characteristics are summarized.

**Table S1** Pilot study TiO_2_ and CeO_2_ aerosol characteristics

|  | TiO_2_ | | | CeO_2_ | | |
| --- | --- | --- | --- | --- | --- | --- |
| NM code | NM-105 | | | NM-212 | | |
| Nominal particle size (nm) | 21.6 ± 1.5 | | | 28.4 ± 10.4 | | |
| Target concentration (mg/m^3^) | 20 | | | 20 | | |
| Gravimetric concentration (mg/m^3^) ± sd | 19.4 | ± | 2.38 | 20.2 | ± | 7.48 |
| Mass concentration TEOM (mg/m^3^) ± sd | 13.7 | ± | 1.65 | 16.5 | ± | 5.51 |
| Particle number concentration (p/cc) ± sd | 1.50E+04 | ± | 6.73E+03 | 3.66E+04 | ± | 1.44E+04 |
| MMAD (µm) ± gsd | 0.79 | ± | 3.04 | 1.06 | ± | 2.32 |
| Count Median Diameter (nm) ± gsd | 234 | ± | 1.72 | 135 | ± | 1.82 |

During exposure of the TiO_2_ control group the relative humidity of the test atmosphere was 49.0 – 51.3 %RH, and the temperature was 22.1 – 22.5 °C. During exposure of the CeO_2_ control group the relative humidity of the test atmosphere was 49.0 – 51.3 %RH, and the temperature was 22.1 – 22.5 °C.

The metal concentrations in the lung compartments and liver tissues were converted to the total metal content based on the dry weight of the tissues or the volume of the cell pellet and BAL fluid.

All blanks had <0.02 mg Ti/kg = <0.02 µg/g. while positive controls with spiked Ti ions and nanoparticle-Ti were respectively 105% and 93%. All controls were according to quality standard. All tissues or fluid were weighed in and Ti content is expressed per microgram.

**Table S2** Pilot Ce concentrations and dose in lavage lung, BALF, BAL cell pellet and liver tissue 18 hours after a single 6 hour exposure to CeO_2_ NM-212.

| **CeO_2_** | **Exposure concentration**  **(mg/m^3^)** | **Lav. lung tissue Ce**  **(µg/g)** | **Lav. lung**  **dry weight (g)** | **Lav. lung tissue dose**  **(µg)** | **BALF Ce**  **(µg /g)** | **BALF dose (µg)** | **BALC Ce**  **(µg/g)** | **BALC dose**  **(µg)** | **Liver Ce**  **(µg/g)** | **Liver dry weight (g)** | **Liver dose (µg)** |
| --- | --- | --- | --- | --- | --- | --- | --- | --- | --- | --- | --- |
| 1 | 0 | <LOD | \| 0.3415 \| \| --- \| | 0.0034 | <LOD | <LOD | <LOD | <LOD | 0.0003 | 4.3606 | 1.17 |
| 2 | 0 | <LOD | 0.3814 | 0.0038 | <LOD | <LOD | <LOD | <LOD | 0.0002 | 4.8682 | 0.73 |
| 3 | 20.2 | 373 | \| 0.3257 \| \| --- \| | **121.4** | 0.142 | **1.455** | 5.304 | **5.328** | 0.001 | 4.7152 | **6.70** |
| 4 | 20.2 | 362 | \| 0.3417 \| \| --- \| | **123.8** | 0.069 | **0.798** | 4.140 | **4.159** | 0.002 | 5.4369 | **9.68** |

< LOD Is below limit of detection

The dose in the lavaged lung tissue is calculated by correcting the concentration for the lavaged lung dry weight. The dose in the BAL fluid is calculated by multiplying the measured concertation to the recovered BALF volume and the density of saline (1.0046 g/cm^3^). The dose in the BAL pellet is calculated by correcting for the density of saline. The total lung dose is calculated by averaging the duplicate measurements and adding the dose in the lavaged lung tissue, BAL fluid and BAL cells. The dose in the liver is calculated by correcting for the total liver dry weight.

**Table S3** Pilot Ti concentrations and dose in lavage lung, BALF, BAL cell pellet and liver tissue 18 hours after a single 6 hour exposure to TiO_2_ NM-105.

| **Animal** | **Exposure concen-tration (mg/m^3^)** | **Lav. lung tissue Ti**  **(µg/g)** | **Lav. lung**  **dry weight (g)** | **Lav. lung tissue dose**  **(µg)** | **BALF Ti**  **(µg/g)** | **BALF dose (µg)** | **BALC Ti**  **(µg/g)** | **BALC dose (µg)** | **Liver Ti**  **(µg/g)** | **Liver dry weight (g)** | **Liver dose (µg)** |
| --- | --- | --- | --- | --- | --- | --- | --- | --- | --- | --- | --- |
| 1 | 0 | 0.27 | 0.315 | 0.09 | <LOD | <LOD | <LOD | <LOD | 0.07 | 4.019 | **Not above control** |
| 2 | 0 | 0.23 | 0.284 | 0.07 | <LOD | <LOD | <LOD | <LOD | 0.05 | 4.870 | **Not above control** |
| 3 | 19.3 | 339 | 0.290 | **98.38** | 0.05 | **0.573** | 1.8 | **1.808** | 0.05 | 4.659 | **Not above control** |
| 4 | 19.3 | 274 | 0.381 | \| **104.45** \| \| --- \| | 0.05 | **0.608** | 4.0 | **4.018** | 0.05 | 4.522 | **Not above control** |

< LOD Is below limit of detection

**Table S4** BALF markers determine 18 hours after a 6 hour exposure to CeO_2_ in the pilot study

| **Animal** | **Exposure concen-tration (mg/m^3^)** | **LDH** **(IU/L)** | **Total protein** **(mg/ml)** | **Total cells**  **(10E^6^/ml)** | **% macrophages** | **% PMNs** | **Particles**  **in macrophages** |
| --- | --- | --- | --- | --- | --- | --- | --- |
| 1 | 0 | < LOD* | 0.44 | 0.508 | 75 | 2.5 | No |
| 2 | 0 | < LOD* | 0.29 | 0.460 | 82 | 0.25 | No |
| 3 | 20.2 | 38 | 0.70 | 0.450 | 79 | 0.5 | Yes |
| 4 | 20.2 | < LOD* | 0.39 | 0.487 | 80 | 1.0 | Yes |

* LOD = 25 IU/L

**Table S5** BALF markers determined 18 hours after a 6 hour exposure to TiO_2_ in the pilot study

| **Animal** | **Exposure concen-tration (mg/m^3^)** | **LDH** **(IU/L)** | **Total protein (mg/ml)** | **Total cells**  **(10E^6^/ml)** | **% macrophages** | **% PMNs** | **Particles**  **in macrophages** |
| --- | --- | --- | --- | --- | --- | --- | --- |
| 1 | 0 | < LOD* | 0.19 | 0.444 | 94 | 1.0 | No |
| 2 | 0 | < LOD* | 0.15 | 0.524 | 76 | 1.3 | No |
| 3 | 19.3 | < LOD* | 0.37 | 0.364 | 86 | 2.5 | Yes |
| 4 | 19.3 | 34 | 0.37 | 0.437 | 89 | 1.25 | Yes |

* LOD = 25 IU/L
