# Supplementary material for: Biodistribution of cerium dioxide and titanium dioxide nanomaterials in rats after single and repeated inhalation exposures
Source: Part Fibre Toxicol. 2024 Aug 14;21:33. doi: 10.1186/s12989-024-00588-4 (PMC11323389; doi:10.1186/s12989-024-00588-4)
Supplement: Supplementary file 2 — Supplementary Material 2 [file 12989_2024_588_MOESM2_ESM.docx]

**Additional file 2 Tissue dose and statistics after single and repeated CeO_2_ exposure**

*Tissue and excreta cerium concentrations converted to dose*

Cerium concentrations in the lung (sum of the BAL cell, BAL fluid and lavage lung tissue compartments), lymph nodes, liver, kidney, spleen, urine and feces have been corrected for the total organ dry weight or total number of ml or grams of excretions to obtain the total cerium content (in microgram per organ or excretion) after a single or repeated exposure to CeO_2_ (Figure S1 and Figure S2).


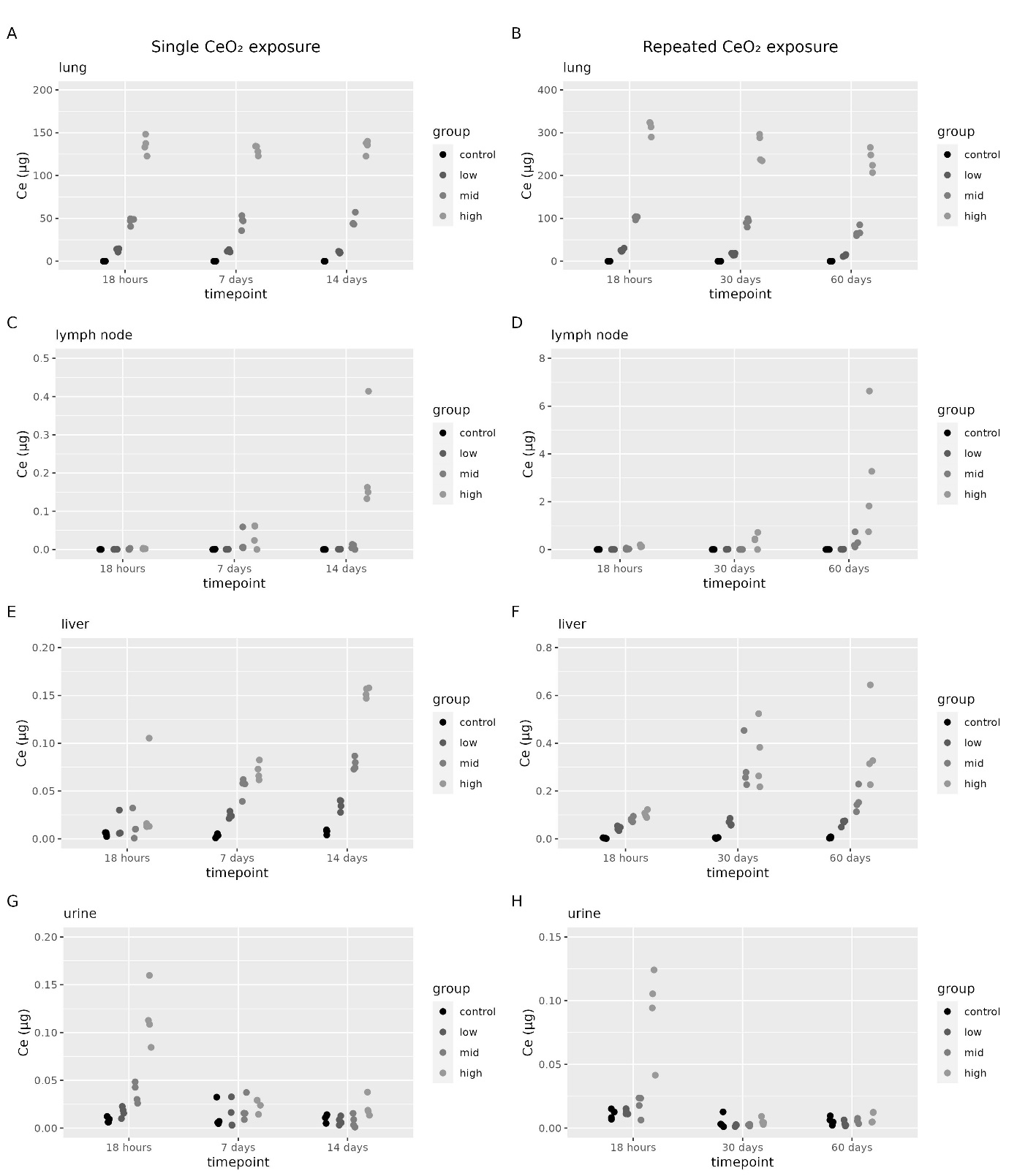


**Figure S1** Cerium dose in microgram (dry organ or total excreted volume in given time period) were assessed 18 hours, 7 days and 14 days after a single (1 day) CeO_2_ exposure. Or at 18 hours, 30 days or 60 days after a repeated (2x5 days) CeO_2_ exposure in control animals and for the low, mid and high exposure groups (n=4 per group) in (A) lungs after a single exposure, (B) lungs after a repeated exposure, (C) mediastinal lymph nodes after a single exposure (D) mediastinal lymph nodes after a repeated exposure, (E) liver after a single exposure, (F) liver after a repeated exposure, (G) urine after a single exposure and (H) urine after a repeated exposure.


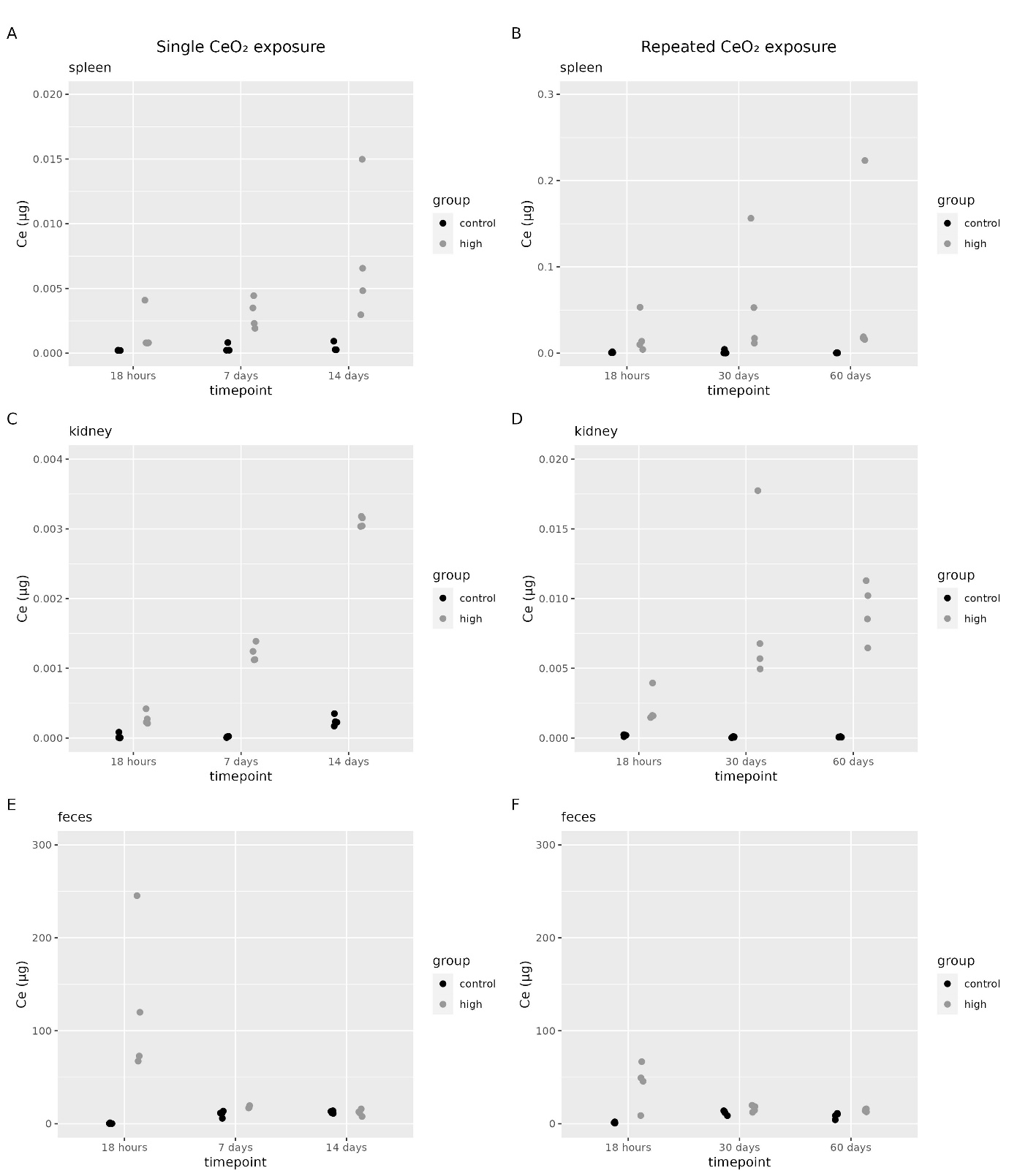


**Figure S2** Cerium dose in microgram (dry organ) was assessed 18 hours, 7 days and 14 days after a single (1 day) CeO_2_ exposure. Or 18 hours, 30 days or 60 days after a repeated (2x5 days) CeO_2_ exposure in control animals and high exposure groups (n=4 per group) in (A) spleen after a single exposure, (B) spleen after a repeated exposure, (C) kidney after a single exposure (D) kidney after a repeated exposure, (E) feces after a single exposure and (F) feces after a repeated exposure.

*Statistics*

Two-way ANOVA on the cerium concentrations in lavaged lung tissue, BAL cell, BAL fluid, mediastinal lymph nodes, liver, spleen, kidney and urine (control, low, mid and high dose) and blood as well as the cerium content in the lung (sum of three compartments), mediastinal lymph nodes, liver, spleen, urine and feces after a single exposure to CeO_2_ NM-212 (Table S1) with a Bonferroni multiple comparison test for those organs/excretions with control versus high dose group only (kidney, spleen, blood and feces) (Table S2).

**Table S1** 2 way ANOVA after single CeO_2_ exposure

| **Parameter** | ***exposure*** | ***time*** | ***exposure*time*** |
| --- | --- | --- | --- |
| Lavaged lung tissue (conc) | *** | ** | n.s. |
| BAL cells (conc) | *** | ** | ** |
| BAL fluid (conc) | *** | *** | *** |
| Total lung (dose) | *** | n.s. | n.s. |
| Lymph nodes (conc) | *** | *** | *** |
| Lymph nodes (dose) | *** | *** | *** |
| Liver (conc) | *** | *** | *** |
| Liver dose | *** | *** | *** |
| Urine (conc) | *** | *** | *** |
| Urine (dose) | *** | *** | *** |
| Kidney (conc) ^a^ | *** | *** | *** |
| Kidney dose ^a^ | *** | *** | *** |
| Spleen (conc) ^a^ | *** | * | * |
| Spleen (dose) ^a^ | ** | n.s. | n.s. |
| Blood (conc) ^a^ | n.s. | n.s. | n.s. |
| Feces (conc) ^a^ | *** | *** | *** |
| Feces (dose) ^a^ | *** | n.s. | *** |

**Table S2** Bonferroni Multiple Comparison test results

| **Parameter** | ***1 day*** | ***7 days*** | ***14 days*** |
| --- | --- | --- | --- |
| Kidney (conc) | n.s. | *** | *** |
| Kidney dose | *** | *** | *** |
| Spleen (conc) | n.s. | n.s. | *** |
| Spleen (dose) | n.s. | n.s. | ** |
| Blood (conc) | n.s. | n.s. | n.s. |
| Feces (conc) | *** | ** | n.s. |
| Feces (dose) | *** | n.s. | n.s. |

| n.s. | no significance |
| --- | --- |
| * | P < 0.05 |
| ** | P < 0.01 |
| *** | P < 0.001 |
| ^a^ | based on control and high dose only |

**Table S3** 2 way ANOVA after repeated (2 x 5 days) CeO_2_ exposure for control, low, mid and high dose group

| **Parameter** | ***exposure*** | ***time*** | ***exposure*time*** |
| --- | --- | --- | --- |
| Lavaged lung tissue (conc) | *** | *** | *** |
| BAL cells (conc) | *** | *** | *** |
| BAL fluid (conc) | *** | *** | *** |
| Total lung (dose) | *** | *** | *** |
| Lymph node (conc) | *** | *** | *** |
| Lymph node (dose) | *** | *** | *** |
| Liver (conc) | *** | ** | * |
| Liver (dose) | *** | *** | *** |
| Urine (conc) | *** | *** | *** |
| Urine (dose) | *** | *** | *** |
| Kidney (conc) ^a^ | *** | n.s. | n.s. |
| Kidney dose ^a^ | *** | * | * |
| Spleen (conc) ^a^ | * | n.s. | n.s. |
| Spleen dose ^a^ | * | n.s. | n.s. |
| Blood (conc) ^a^ | * | n.s. | n.s. |
| Feces (conc) ^a^ | *** | *** | *** |
| Feces (dose) ^a^ | *** | n.s. | *** |

**Table S4** Bonferroni Multiple Comparison test results for control and high dose group

| **Parameter** | ***1 day*** | ***30 days*** | ***60 days*** |
| --- | --- | --- | --- |
| Kidney (conc) | n.s. | *** | *** |
| Kidney dose | n.s. | *** | *** |
| Spleen (conc) | n.s. | n.s. | n.s. |
| Spleen dose | n.s. | n.s. | n.s. |
| Blood (conc) | n.s. | n.s. | * |
| Feces (conc) | *** | n.s. | n.s. |
| Feces (dose) | *** | n.s. | n.s. |

| n.s. | no significance |
| --- | --- |
| * | P < 0.05 |
| ** | P < 0.01 |
| *** | P < 0.001 |
| ^a^ | based on control and high dose only |
