# Supplementary material for: Biodistribution of cerium dioxide and titanium dioxide nanomaterials in rats after single and repeated inhalation exposures
Source: Part Fibre Toxicol. 2024 Aug 14;21:33. doi: 10.1186/s12989-024-00588-4 (PMC11323389; doi:10.1186/s12989-024-00588-4)
Supplement: Supplementary file 3 — Supplementary Material 3 [file 12989_2024_588_MOESM3_ESM.docx]

**Additional file 3 Tissue dose and statistics after single and repeated TiO_2_ exposure**

*Tissue and excreta titanium concentrations converted to dose*

Titanium concentrations in the lung (sum of the BAL cell, BAL fluid and lavage lung tissue compartments), liver, kidney, spleen, urine and feces have been corrected for the total organ dry weight or total number of ml or grams of excretions to obtain the total titanium content (reported in microgram per organ or excretion) after a single or repeated exposure to TiO_2_ (Figure S1 and Figure S2).


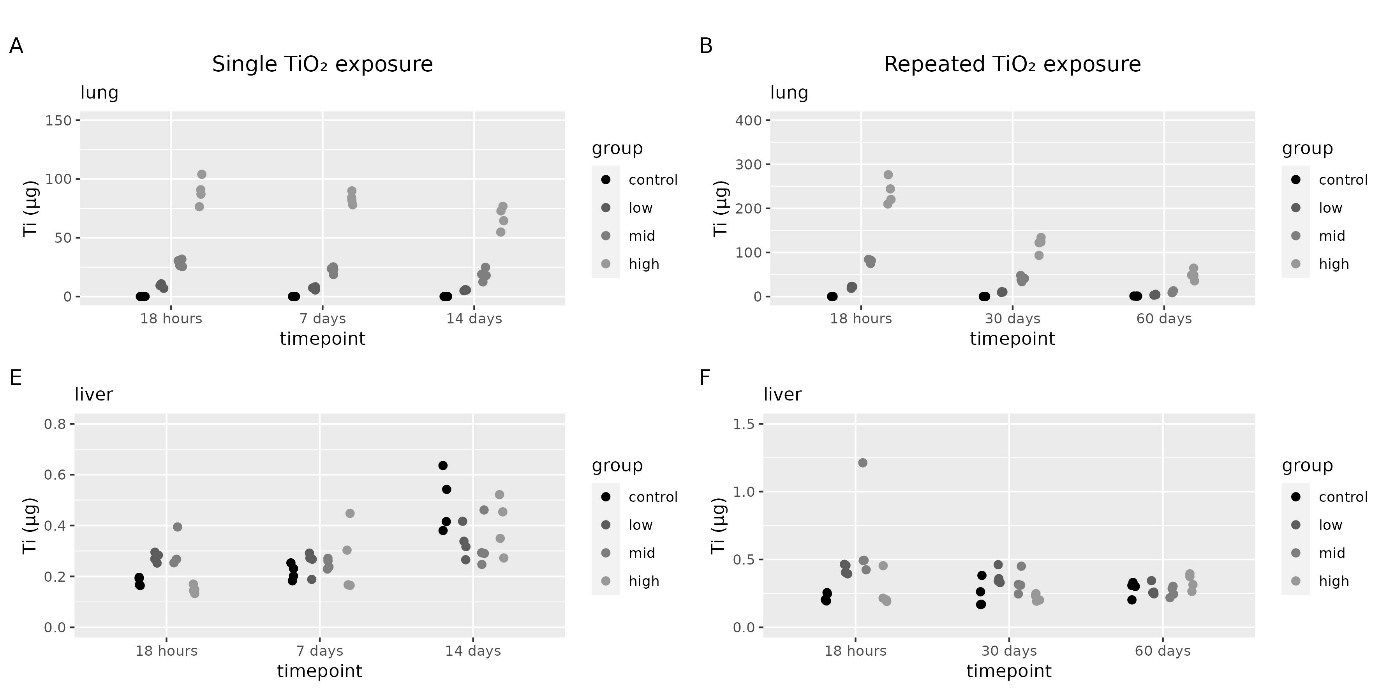


**Figure S1** Titanium dose in microgram (dry organ) was assessed 18 hours, 7 days and 14 days after a single (1 day) TiO_2_ exposure. Or at 18 hours, 30 days or 60 days after a repeated (2x5 days) TiO_2_ exposure in control animals and for the low, mid and high exposure groups (n=4 per group) in (A) lungs after a single exposure, (B) lungs after a repeated exposure, (C) liver after a single exposure (D) liver after a repeated exposure.


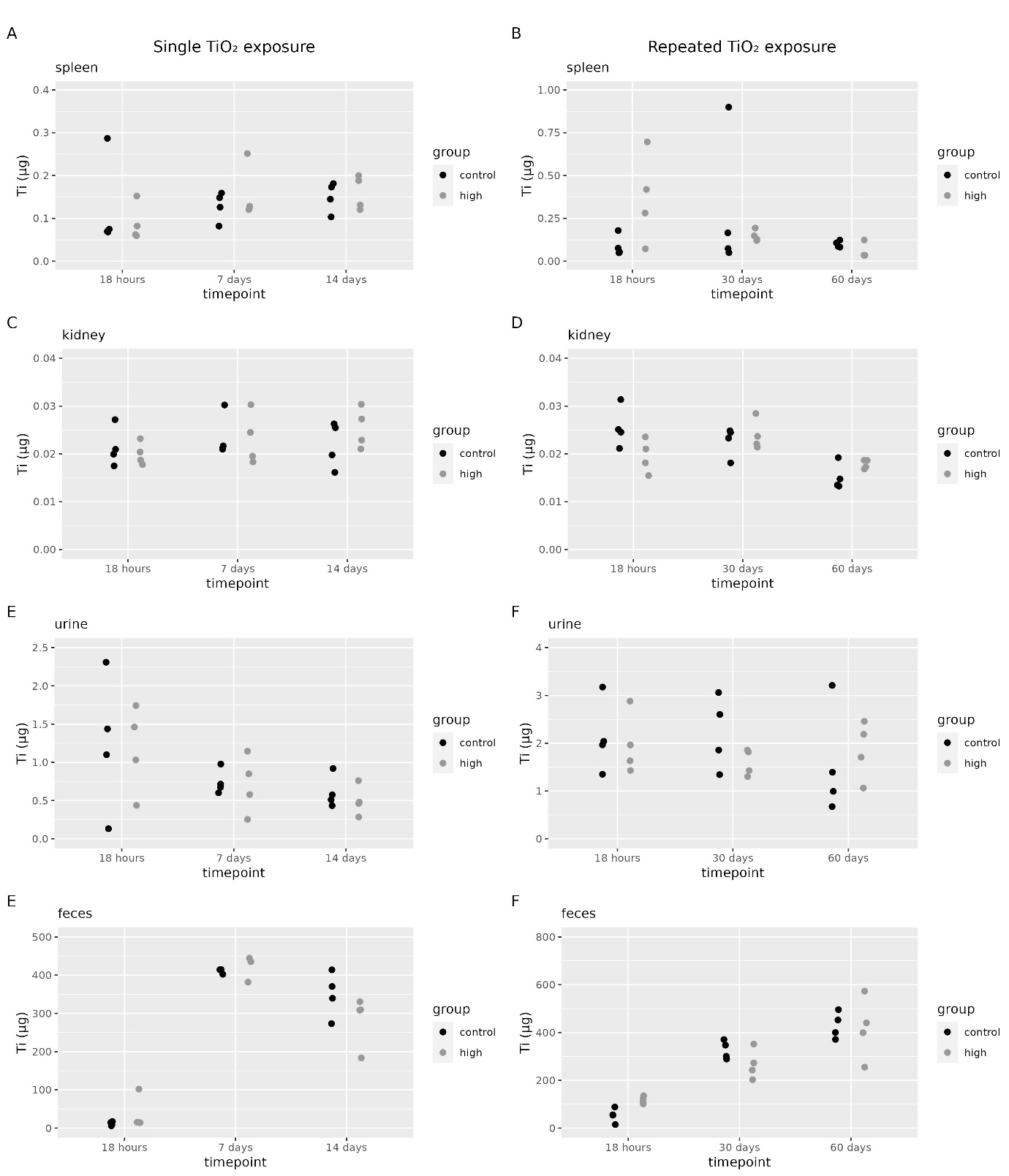


**Figure S2** Titanium dose in microgram (dry organ) was assessed 18 hours, 7 days and 14 days after a single (1 day) TiO_2_ exposure. Or 18 hours, 30 days or 60 days after a repeated (2x5 days) TiO_2_ exposure in control animals and high exposure groups (n=4 per group) in (A) spleen after a single exposure, (B) spleen after a repeated exposure, (C) kidney after a single exposure (D) kidney after a repeated exposure, (E) urine after a single exposure, (F) urine after a repeated exposure, (G) feces after a single exposure and (H) feces after a repeated exposure.

*Statistics*

Two-way ANOVA on the titanium concentrations in lavaged lung tissue, BAL cell, BAL fluid, mediastinal lymph nodes, liver, spleen, kidney and urine (control, low, mid and high dose) and blood as well as the titanium content in the lung (sum of three compartments), mediastinal lymph nodes, liver, spleen, urine and feces after a single exposure to TieO_2_ NM-212 (Table S1) with a Bonferroni multiple comparison test for those organs/excretions with control versus high dose group only (kidney, spleen, blood and feces) (Table S2).

**Table S1** 2 way ANOVA after a single TiO_2_ exposure for control, low, mid and high dose group

| **Parameter** | ***exposure*** | ***time*** | ***interactie*** |
| --- | --- | --- | --- |
| Lavaged lung tissue (conc) | *** | *** | n.s. |
| BAL cells (conc) | *** | * | n.s. |
| BAL fluid (conc) | n.s. | n.s. | n.s. |
| Total lung (dose) | *** | *** | * |
| Lymph nodes (conc) | ** | ** | n.s. |
| Liver (conc) | n.s. | *** | * |
| Liver (dose) | n.s. | *** | ** |
| Blood | ****** | ****** | ***** |
| Kidney (conc) ^a^ | n.s. | n.s. | n.s. |
| Kidney dose ^a^ | n.s. | n.s. | n.s. |
| Spleen (conc)  ^a^ | n.s. | n.s. | n.s. |
| Spleen dose ^a^ | n.s. | n.s. | n.s. |
| Urine (conc) ^a^ | n.s. | ** | n.s. |
| Urine dose ^a^ | n.s. | n.s. | n.s. |
| Feces (conc) ^a^ | *** | *** | *** |
| Feces (dose) ^a^ | n.s. | *** | n.s. |

**Table S2** Bonferroni Multiple Comparison test results control versus high dose group

| **Parameter** | ***1 day*** | ***7 days*** | ***14 days*** |
| --- | --- | --- | --- |
| Kidney (conc) | n.s. | n.s. | n.s. |
| Kidney dose) | n.s. | n.s. | n.s. |
| Spleen (conc) | n.s. | n.s. | n.s. |
| Spleen dose | n.s. | n.s. | n.s |
| Urine (conc) | n.s. | n.s. | n.s. |
| Urine (dose) | n.s. | n.s. | n.s. |
| Feces (conc) | *** | n.s. | n.s. |
| Feces (dose) | n.s. | n.s. | n.s. |

| n.s. | no significance |
| --- | --- |
| * | P < 0.05 |
| ** | P < 0.01 |
| *** | P < 0.001 |
| ^a^ | based on control and high dose only |

**Table S3** 2 way ANOVA after repeated (2 x 5 days) TiO_2_ exposure

| **Parameter** | ***exposure*** | ***time*** | ***interactie*** |
| --- | --- | --- | --- |
| Lavaged lung tissue (conc) | *** | *** | *** |
| BALF cells (conc) | *** | * | n.s. |
| BALF (conc) | ** | n.s. | ** |
| Total lung (dose) | *** | *** | *** |
| Lymph nodes (conc) | *** | ** | * |
| Liver (conc) | * | n.s. | * |
| Liver (dose) | ** | * | ** |
| Blood | * | *** | n.s. |
| Kidney (conc) ^a^ | n.s. | ******* | ****** |
| Kidney dose ^a^ | n.s. | *** | * |
| Spleen (conc) ^a^ | n.s. | n.s. | n.s. |
| Spleen dose ^a^ | n.s. | n.s. | n.s. |
| Urine (conc) ^a^ | n.s. | *** | n.s. |
| Urine dose | n.s. | n.s. | n.s. |
| Feces (conc) ^a^ | n.s. | *** | n.s. |
| Feces (dose) ^a^ | n.s. | *** | ** |

**Table S4** Bonferroni Multiple Comparison test results (for parameters with only control and high dose group).

| **Parameter** | ***1 day*** | ***30 days*** | ***60 days*** |
| --- | --- | --- | --- |
| kidney (conc) | ** | n.s. | n.s. |
| Kidney dose | * | n.s. | n.s. |
| Spleen (conc) | n.s. | n.s. | n.s. |
| Spleen dose | n.s. | n.s. | n.s. |
| Urine (conc) | n.s. | n.s. | n.s. |
| Urine dose | n.s. | n.s. | n.s. |
| Feces (conc) | n.s. | n.s. | n.s. |
| Feces (dose) | ** | n.s. | n.s. |

| n.s. | no significance |
| --- | --- |
| * | P < 0.05 |
| ** | P < 0.01 |
| *** | P < 0.001 |
| ^a^ | based on control and high dose only |
