# Supplementary material for: Biodistribution of cerium dioxide and titanium dioxide nanomaterials in rats after single and repeated inhalation exposures
Source: Part Fibre Toxicol. 2024 Aug 14;21:33. doi: 10.1186/s12989-024-00588-4 (PMC11323389; doi:10.1186/s12989-024-00588-4)
Supplement: Supplementary file 4 — Supplementary Material 4 [file 12989_2024_588_MOESM4_ESM.docx]

**Additional file 4 Toxicological assessment in BALF**

**Table S1** BALF cytology and total protein data were log transformed and analyzed by two-way ANOVA.

| parameter | ***exposure*** | ***time*** | ***exposure*time*** |
| --- | --- | --- | --- |
| 1d Ce protein | n.s. | n.s. | n.s. |
| 1d Ce Total Cells BALF | *** | * | ** |
| 1d Ce Macrophages | ** | n.s. | n.s. |
| 1d Ce Neutrophils | ***  ^1^ | ***  ^1^ | *** ^1^ |
| 2x5d Ce protein | * | ** | n.s. |
| 2x5d Ce Total Cells BALF | *** | n.s. | n.s. |
| 2x5d Ce Macrophages | * | n.s. | n.s. |
| 2x5d Ce Neutrophils | *** | *** | *** |
| 1d Ti protein | n.s. | * | ** |
| 1d Ti Total Cells BALF | n.s. | * | n.s. |
| 1d Ti Macrophages | n.s. | * | n.s. |
| 1d Ti Neutrophils | n.s. | n.s. | n.s. |
| 2x5d Ti protein | * | *** | ** |
| 2x5d Ti Total Cells BALF | n.s. | n.s. | n.s. |
| 2x5d Ti Macrophages | n.s. | n.s. | n.s. |
| 2x5d Ti Neutrophils | * | * | ** |

1d Ce or Ti is referring to the single exposure to CeO_2_ or TiO_2_

2x5 d Ce or Ti is referring to a repeated exposure to CeO_2_ or TiO_2_

1. Significance only driven by high dose group on day 14.

| n.s. | no significance |
| --- | --- |
| * | P < 0.05 |
| ** | P < 0.01 |
| *** | P < 0.001 |
