# Supplementary material for: Biodistribution of cerium dioxide and titanium dioxide nanomaterials in rats after single and repeated inhalation exposures
Source: Part Fibre Toxicol. 2024 Aug 14;21:33. doi: 10.1186/s12989-024-00588-4 (PMC11323389; doi:10.1186/s12989-024-00588-4)
Supplement: Supplementary file 5 — Supplementary Material 5 [file 12989_2024_588_MOESM5_ESM.docx]

**Additonal file 5**

X-ray diffraction analysis confirmed the crystal structure for TiO_2_ NM-105 as anatase 86.3 ± 0.2 : Rutile 13.7 ± 0.1 as expected.

**Figure S1** XRD analysis
