# Supplementary figures and images for: Biodistribution of cerium dioxide and titanium dioxide nanomaterials in rats after single and repeated inhalation exposures
Source: Part Fibre Toxicol. 2024 Aug 14;21:33. doi: 10.1186/s12989-024-00588-4 (PMC11323389; doi:10.1186/s12989-024-00588-4)

**Additional file 6**

**
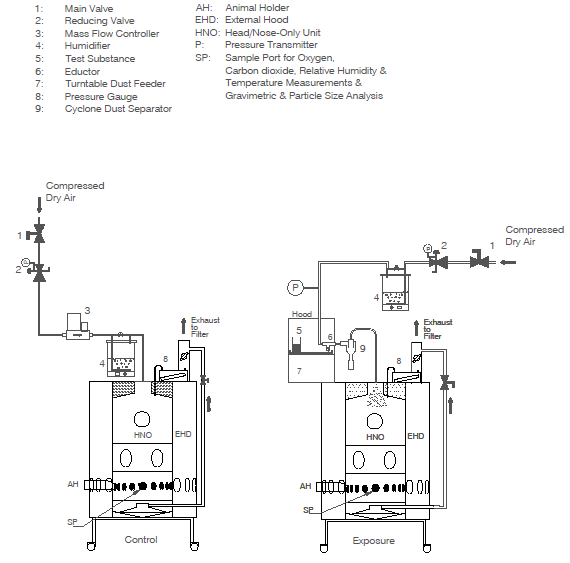
**

**Figure S1** Drawing of test atmosphere generation set-up

Supplement: Supplementary file 6 — Supplementary Material 6 [file 12989_2024_588_MOESM6_ESM.docx]
