# Supplementary material for: Biodistribution of cerium dioxide and titanium dioxide nanomaterials in rats after single and repeated inhalation exposures
Source: Part Fibre Toxicol. 2024 Aug 14;21:33. doi: 10.1186/s12989-024-00588-4 (PMC11323389; doi:10.1186/s12989-024-00588-4)
Supplement: Supplementary file 7 — Supplementary Material 7 [file 12989_2024_588_MOESM7_ESM.docx]

**Additional file 7 Sample preparation for ICP-MS measurements**

**Table S1** Sample preparation procedure for the digestion of rat samples for cerium ICP-MS measurements.

| **Sample** | **Sample weight used for digestion** | **Added reagents** | **Weight of the sample digest (g)** | **Sample dilution prior ICP-MS analysis** |
| --- | --- | --- | --- | --- |
| Lung tissue (freeze dried) | Whole sample (corresponding to 0.27-0.55 g, dry weight) | 3.2 ml conc. HNO_3_ + 0.8 mL UPW | 40 | 200x |
| BAL fluid (liquid) | 0.2 g | 0.8 ml conc. HNO_3_ | 10 | 2.5x |
| BAL cell suspension (liquid) | 0.1 g | 0.8 ml conc. HNO_3_ | 10 | 20x |
| Lung lymph nodes (frozen) | Whole sample (corresponding to 1.5-26.3 mg, wet weight) | 0.5 ml conc. HNO_3_ + 0.1 mL UPW | 10 | 1.56x |
| Liver (freeze dried) | 0.1 g, dry weight | 0.8 ml conc. HNO_3_ + 0.2 mL UPW | 10 | 2.5x |
| Spleen (freeze dried) | Whole sample (corresponding to 0.12-0.22 g, dry weight) | 1.6 ml conc. HNO_3_ + 0.4 mL UPW | 20 | 2.5x |
| Kidney (freeze dried) | Whole sample split into two sub-samples (corresponding to 0.25-0.43 g, dry weight) | 3.2 ml conc. HNO_3_ + 0.8 mL UPW | 40 | 2.5x |
| Urine (liquid) | 0.5 g | 0.8 ml conc. HNO_3_ | 10 | 2.5x |
| Faeces (freeze dried) | 0.1 g, dry weight | 4 ml conc. HNO_3_ | 25 | 40x |
| Blood (liquid) | 0.5 g | 2 ml conc. HNO_3_ | 25 | 2.5x |
| Food pellets | 0.05 g | 6 ml conc. HNO_3_ | 25 | 7.5x |

**Table S2** Preparation of the spiked samples for cerium ICP-MS measurements

| **Sample** | **Sample used for spiking** | **Spiking concentration** |
| --- | --- | --- |
| Lung tissue (freeze dried) | Liver tissue from control experiment | 250 µg/g |
| BAL fluid (liquid) | BAL fluid from control experiment | 100 ng/g |
| BAL cell suspension (liquid) | BAL cell suspension from control experiment | 2 µg/g |
| Lung lymph nodes (frozen) | Liver tissue from control experiment | 20 ng/g |
| Liver (freeze dried) | Liver tissue from control experiment | 5 ng/g |
| Spleen (freeze dried) | Liver tissue from control experiment | 20 ng/g |
| Kidney (freeze dried) | Liver tissue from control experiment | 20 ng/g |
| Urine (liquid) | Urine from control experiment | 5 ng/g |
| Faeces (freeze dried) | Faeces from control experiment | 25 µg/g |
| Blood (liquid) | Blood from control experiment | 5 ng/g |
| Food pellets | Food pellets | 500 ng/g |

**Table S3** Instrumental settings for ICP-MS analysis cerium

| **Parameter** | **Value/type** |
| --- | --- |
| RF Power | 1550 W |
| RF Matching | 1.80 V |
| Sampling depth | 8.0 mm |
| Plasma gas flow rate | 15 L/min |
| Nebulizer gas flow rate | 1.06 L/min |
| Auxiliary gas flow rate | 0.90 L/min |
| Monitored isotope | ^140^Ce |
| Isotope of internal standard | ^103^Rh |
| Dwell time | 0.3 s (Ce), 0.1 s (Rh) |
| Nebulizer type | Micromist, concentric |
| Spray chamber | Scott type (double-pass, quartz) |
| Torch, id injector | - 1. mm |

**Table S4** Estimated LOD and LOQ values for mass concentration of cerium in different rat samples determined by ICP-MS.

| **Sample** | **LOD (ng/g= µg/kg)** | **LOQ (ng/g= µg/kg)** |
| --- | --- | --- |
| Lung tissue | 1.93 – 4.58* | 6.44 – 15.3* |
| BAL fluid | 0.52 | 1.72 |
| BAL cell suspension | 2.08 | 6.94 |
| Lung lymph nodes | 4.9 | 16.5 |
| Liver | 0.14 – 0.31* | 0.46 – 1.04* |
| Spleen | 0.82 | 2.72 |
| Kidney | 0.08 | 0.25 |
| Urine | 0.06 – 0.13* | 0.20 – 0.43* |
| Faeces | 5.3 | 17.5 |
| Blood | 0.06 | 0.19 |
| Food pellets | 0.38 | 1.27 |

* For samples that were analyzed on different days, different LOD/LOQ values were determined.

The trueness of the method was determined as a recovery (%), calculated as the ratio between the determined and expected Ce mass concentration in the spiked samples (summarized in Table 5). The recoveries for Ce mass concentration for all sample types and at different spiking levels were found to be satisfactory (in the range of 92 and 115 %).

**Table S5** Recovery for cerium mass concentration in the spiked samples (N = 1 – 5), determined by ICP-MS.

| **Sample** | **Mass recovery (%)** |
| --- | --- |
| Lung tissue | 96 - 106 |
| BAL fluid | 101 - 103 |
| BAL cell suspension | 106 - 108 |
| Lung lymph nodes | 99 - 115 |
| Liver | 95 - 98 |
| Spleen | 95 - 96 |
| Kidney | 92 - 105 |
| Urine | 95 - 96 |
| Faeces | 100 - 1  14 |
| Blood | 101 |
| Food pellets | 98 |

**Table S6** LOD titanium depends on the sample intake

| **Sample** | **LOD (mg/kg)** | **LOQ (mg/kg)** |
| --- | --- | --- |
| Liver tissue/blood/Spleen  Lung tissue/kidney/faeces  Food pellets/urine | 0.03 | 0.05 |
| Lung lymph nodes | 1 | 2 |
| BAL cell suspension | 0.1 | 0.3 |

**Table S7** Titanium and cerium content in feed pellets in duplo

| **Feed pellets** | **Titanium (µg/g)** | **Cerium (µg/g)** |
| --- | --- | --- |
| 1 | 29 | 0.30 |
| 2 | 26 | 0.24 |

Control samples (empty tubes) were below the limit of detection
