# Supplementary material for: Biodistribution of cerium dioxide and titanium dioxide nanomaterials in rats after single and repeated inhalation exposures
Source: Part Fibre Toxicol. 2024 Aug 14;21:33. doi: 10.1186/s12989-024-00588-4 (PMC11323389; doi:10.1186/s12989-024-00588-4)
Supplement: Supplementary file 8 — Supplementary Material 8 [file 12989_2024_588_MOESM8_ESM.docx]

**Additional file 8**

**Method description lung elimination half life estimation**

For each material and exposure group separately, a linear regression model of the log of the total lung load against the post-exposure time (in days) was fitted. In case of a linear clearance kinetics of the lung, a straight line (linear model) is expected to provide a good description. In that case, the coefficient of the linear model is interpreted as the rate of elimination k (per day) of the lung.

The estimate of the regression coefficient k can be converted to an estimate of the elimination half-life time (day) by

$$t_{1/2}=\ln\left( 2 \right)/k$$

The R function lm() from the R stats package was used (*R Core Team (2020). R: A language and environment for statistical computing. R Foundation for Statistical Computing, Vienna, Austria. URL* [*https://www.R-project.org/*](https://www.R-project.org/)*).*

Statistical significance of the elimination was evaluated using the reported probabilities of the linear regression model for the coefficient. This expressed the probability finding the estimated rate of elimination in case that the elimination, in fact would be zero.

**Results**

The estimated lung elimination half life times in days after CeO_2_ and TiO_2_ exposure are presented in table S1. Y indicates yes, there is significant elimination. N means no significant elimination.

**Table S1** Estimated half life and coefficient of variation after a single and repeated inhalation of CeO_2_ or TiO_2_ ENM aerosol.

| **CeO_2_** | | | | |
| --- | --- | --- | --- | --- |
|  | Halflife (day) | Coefficient of variation | P (t > 0) | Elimination Significantly >0 |
| 1d low | 39.5 | 0.33 | 1.4E-02 | y |
| 1d mid | 6901.0 | 71 | 9.9E-01 | n |
| 1d high | 1144.3 | 5.6 | 8.6E-01 | n |
| 2x5d low | 58.3 | 0.16 | 7.5E-05 | y |
| 2x5d mid | 98.8 | 0.18 | 2.5E-04 | y |
| 2x5d high | 133.7 | 0.23 | 1.3E-03 | Y |
| **TiO_2_** | | | | |
| 1d low | 18.3 | 0.21 | 7.7E-04 | y |
| 1d mid | 19.8 | 0.29 | 5.2E-03 | y |
| 1d high | 30.8 | 0.28 | 5.3E-03 | y |
| 2x5d low | 24.7 | 0.082 | 3.7E-09 | y |
| 2x5d mid | 20.1 | 0.094 | 5.2E-08 | y |
| 2x5d high | 25.7 | 0.090 | 1.6E-07 | y |
