# Supplementary material for: Biodistribution of cerium dioxide and titanium dioxide nanomaterials in rats after single and repeated inhalation exposures
Source: Part Fibre Toxicol. 2024 Aug 14;21:33. doi: 10.1186/s12989-024-00588-4 (PMC11323389; doi:10.1186/s12989-024-00588-4)
Supplement: Supplementary file 9 — Supplementary Material 9 [file 12989_2024_588_MOESM9_ESM.docx]

**Additional file 9**

**Dose-response analysis for toxicological markers after a single and repeated exposure to CeO_2_ and TiO_2_**

### CeO_2_, total cell count


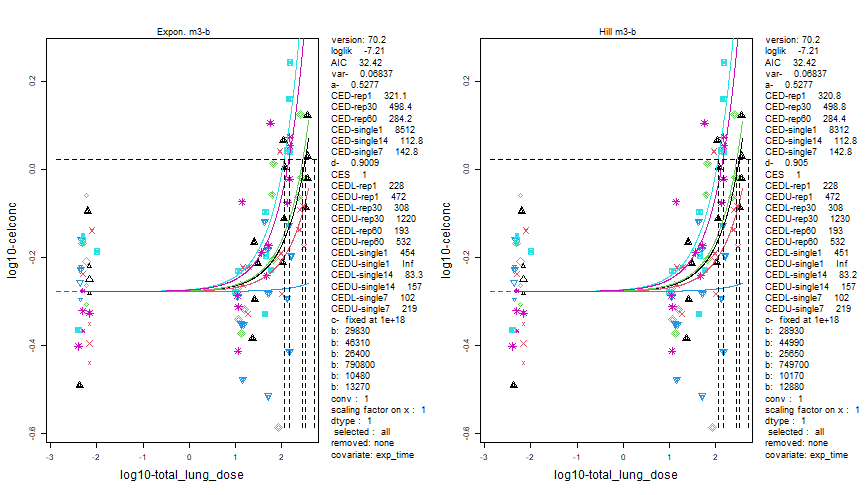


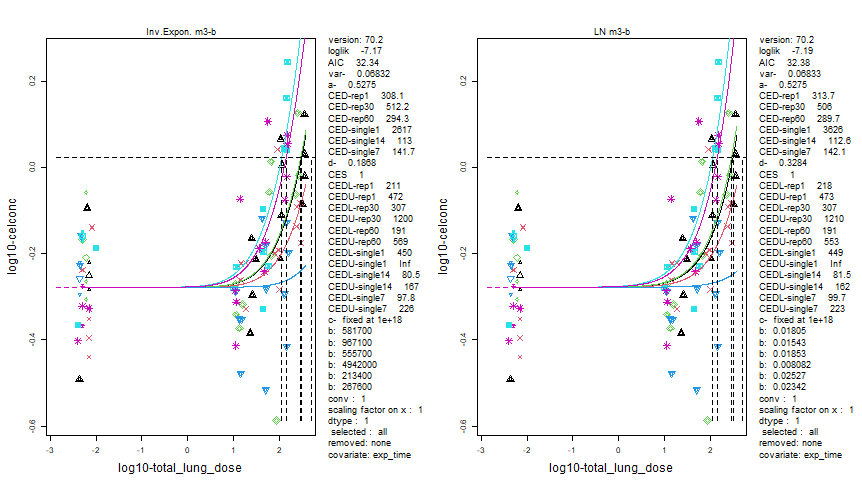


**Figure S1** Four continuous dose-response models (top: exponential and Hill, bottom: inverse exponential and lognormal) fitted to the total cell concentration in BALF (y-axis, 10^6^/L). Dose on the x-axis is the CeO_2_ lung dose (µg). Data of 6 exposure regimes are simultaneously fitted using covariate analysis (EFSA, 2017). Blue downward triangle: 18 hours after single dosing (single1). Pink cross-plus: 7 days after single dosing (single7). Light blue cross-square: 14 days after single dosing (single14). Black triangle: 18 hours after repeated dosing (rep1). Red cross: 30 days after repeated dosing (rep30). Green diamond: 60 days after repeated dosing (rep60). Horizontal dashed line indicates the BMR at 100% change in response compared to background. Vertical dashed lines indicate the BMDs of each exposure regime.


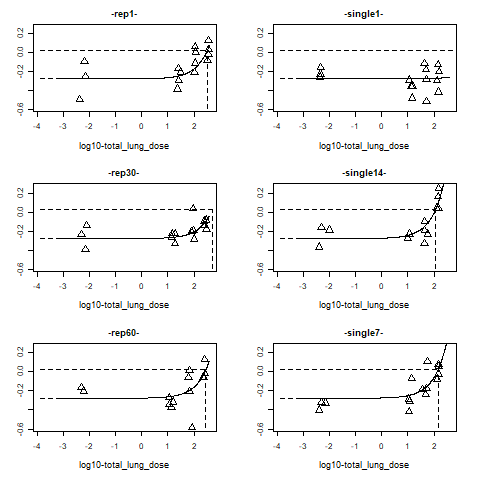


**Figure S2** Data and exponential model fits obtained in figure 1 (top left) now plotted separately for each exposure regime, indicated above each plot.

The weights used in model averaging are exponential: 0.2462, Hill: 0.2462, inverse exponential: 0.2563 and lognormal: 0.2512

The model-average BMD confidence interval is:

| subgroup | BMDL (µg) | BMDU (µg) |
| --- | --- | --- |
| rep1 (18 hours after repeated dosing) | 195 | 4.81E+02 |
| rep30 (30 days after repeated dosing) | 268 | 1.21E+03 |
| rep60 (60 days after repeated dosing) | 163 | 6.17E+02 |
| single1 (18 hours after single dosing) | 327 | Inf |
| single7 (7 days after single dosing) | 84 | 2.64E+02 |
| single14 (14 days after single dosing) | 58.5 | 1.92E+02 |

### CeO2, fraction macrophages


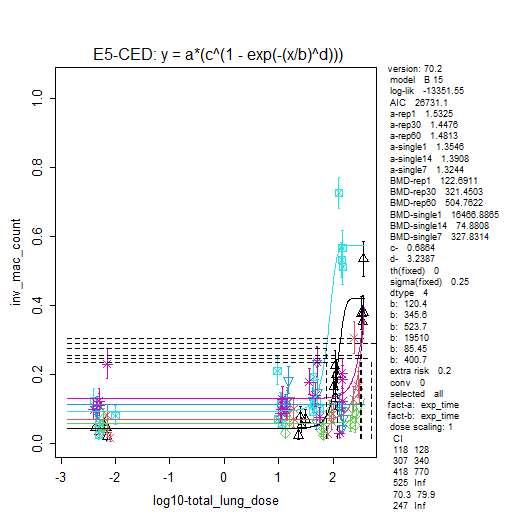


**Figure S3** Exponential dose-response model fitted to the inverse fraction of macrophage count in BALF (y-axis). Dose on the x-axis is the CeO_2_ lung dose (µg). Data of 6 exposure regimes are simultaneously fitted using covariate analysis. Blue downward triangle: 18 hours after single dosing (single1). Pink cross-plus: 7 days after single dosing (single7). Light blue cross-square: 14 days after single dosing (single14). Black triangle: 18 hours after repeated dosing (rep1). Red cross: 30 days after repeated dosing (rep30). Green diamond: 60 days after repeated dosing (rep60). Horizontal dashed line indicates the BMR at 20% extra risk. Vertical dashed lines indicate the BMDs of each exposure regime.


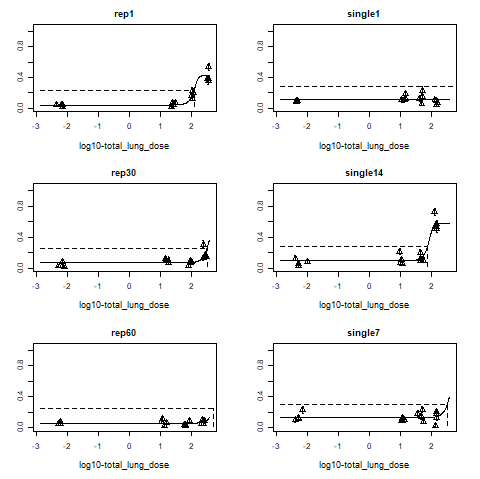


**Figure S4** Data and exponential model fits obtained in figure 3 now plotted separately for each exposure regime, indicated above each plot.

The BMD confidence interval is:

| subgroup | BMDL (µg) | BMDU (µg) |
| --- | --- | --- |
| rep1 (18 hours after repeated dosing) | 118 | 128 |
| rep30 (30 days after repeated dosing) | 307 | 340 |
| rep60 (60 days after repeated dosing) | 418 | 770 |
| single1 (18 hours after single dosing) | 525 | Inf |
| single7 (7 days after single dosing) | 247 | Inf |
| single14 (14 days after single dosing) | 70.3 | 79.9 |

### CeO2, fraction PMN


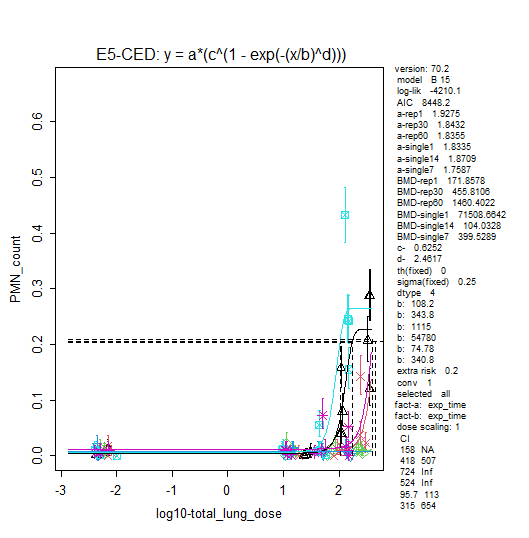


**Figure S5** Exponential dose-response model fitted to the PMN count in BALF (y-axis). Dose on the x-axis is the CeO_2_ lung dose (µg). Data of 6 exposure regimes are simultaneously fitted using covariate analysis. Blue downward triangle: 18 hours after single dosing (single1). Pink cross-plus: 7 days after single dosing (single7). Light blue cross-square: 14 days after single dosing (single14). Black triangle: 18 hours after repeated dosing (rep1). Red cross: 30 days after repeated dosing (rep30). Green diamond: 60 days after repeated dosing (rep60). Horizontal dashed line indicates the BMR at 20% extra risk. Vertical dashed lines indicate the BMDs of each exposure regime.


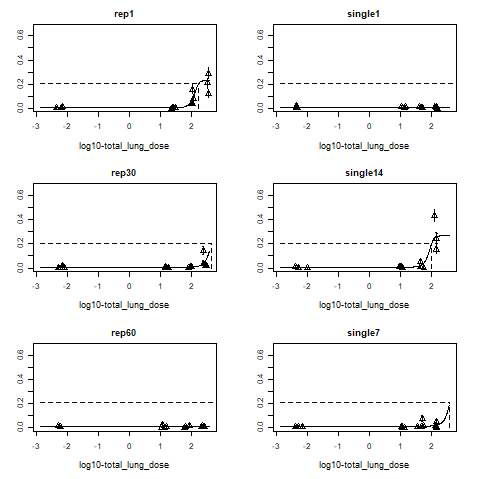


**Figure S6** Data and exponential model fits obtained in figure 5 now plotted separately for each exposure regime, indicated above each plot.

The BMD confidence interval is:

| subgroup | BMDL (µg) | BMDU (µg) |
| --- | --- | --- |
| rep1 (18 hours after repeated dosing) | 158 | Inf |
| rep30 (30 days after repeated dosing) | 418 | 507 |
| rep60 (60 days after repeated dosing) | 724 | Inf |
| single1 (18 hours after single dosing) | 524 | Inf |
| single7 (7 days after single dosing) | 315 | 654 |
| single14 (14 days after single dosing) | 95.7 | 113 |

### CeO_2_, total protein


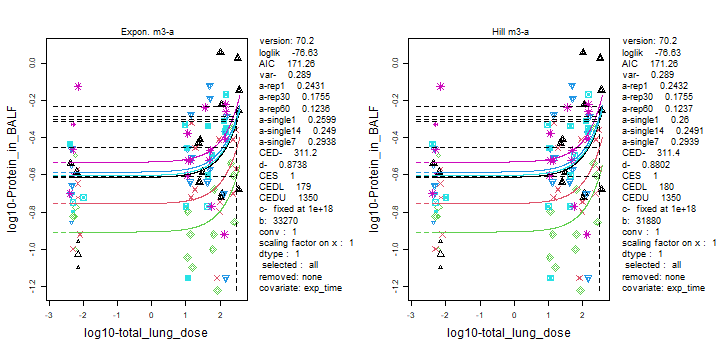


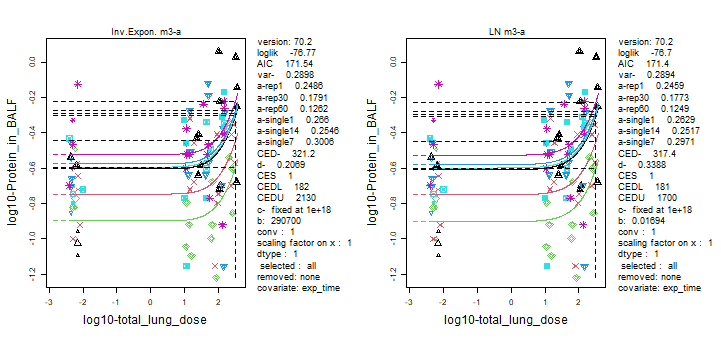


**Figure S7** Four continuous dose-response models (top: exponential and Hill, bottom: inverse exponential and lognormal) fitted to the total protein concentration in BALF (y-axis, g/L). Dose on the x-axis is the CeO_2_ lung dose (µg). Data of 6 exposure regimes are simultaneously fitted using covariate analysis. Blue downward triangle: 18 hours after single dosing (single1). Pink cross-plus: 7 days after single dosing (single7). Light blue cross-square: 14 days after single dosing (single14). Black triangle: 18 hours after repeated dosing (rep1). Red cross: 30 days after repeated dosing (rep30). Green diamond: 60 days after repeated dosing (rep60). Horizontal dashed line indicates the BMR at 100% change in response compared to background. Vertical dashed lines indicate the BMDs of each exposure regime.


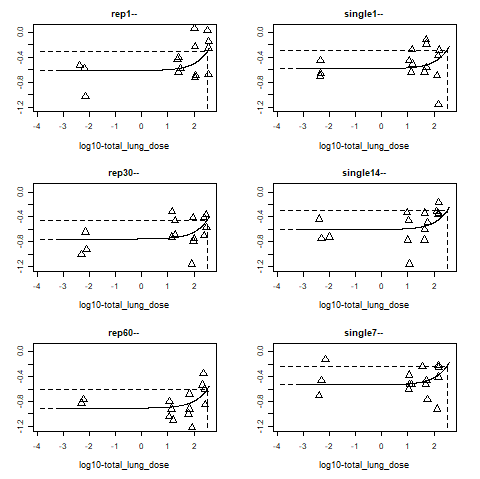


**Figure S8** Data and exponential model fits obtained in figure 7 (top left) now plotted separately for each exposure regime, indicated above each plot.

The weights used in model averaging are exponential: 0.2630, Hill: 0.2630, inverse exponential: 0.2287 and lognormal: 0.2453.

The model-average BMD confidence interval is:

| subgroup | BMDL (µg) | BMDU (µg) |
| --- | --- | --- |
| rep1 (18 hours after repeated dosing) | 132 | 893 |
| rep30 (30 days after repeated dosing) | 118 | 675 |
| rep60 (60 days after repeated dosing) | 112 | 692 |
| single1 (18 hours after single dosing) | 65.7 | 834 |
| single7 (7 days after single dosing) | 55.8 | Inf |
| single14 (14 days after single dosing) | 112 | Inf |

### TiO_2_, total cell count


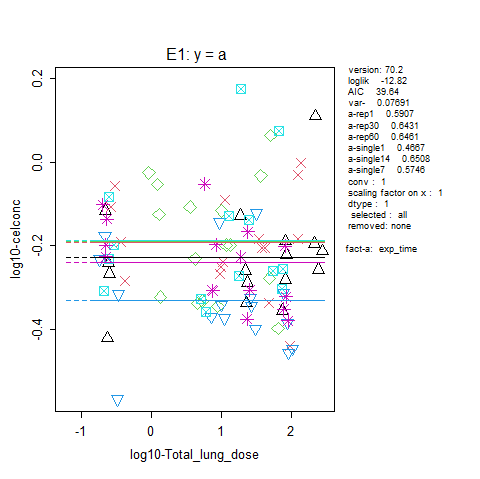


**Figure S9** The four continuous dose-response models (exponential, Hill, inverse exponential and lognormal) fitted to the total cell concentration in BALF (y-axis, 10^6^/L) all showed that there is no trend in the available data. Dose on the x-axis is the TiO_2_ lung dose (µg). Data of 6 exposure regimes are simultaneously fitted using covariate analysis. Blue downward triangle: 18 hours after single dosing (single1). Pink cross-plus: 7 days after single dosing (single7). Light blue cross-square: 14 days after single dosing (single14). Black triangle: 18 hours after repeated dosing (rep1). Red cross: 30 days after repeated dosing (rep30). Green diamond: 60 days after repeated dosing (rep60).

Due to the absence of a trend no BMD confidence interval is derived.

### TiO_2_, fraction macrophages


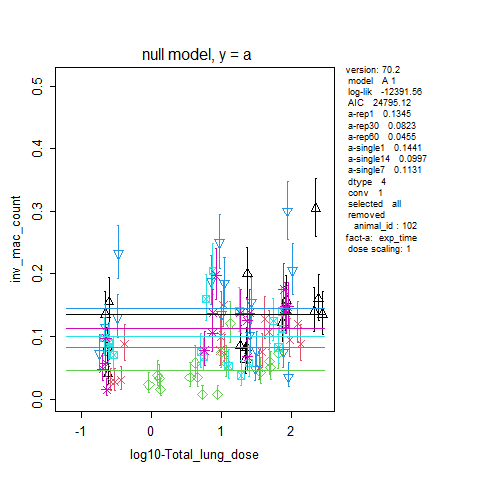


**Figure S10** The exponential latent variable model fitted to the (inverse) macrophage fraction in BALF (y-axis) showed that there is no trend in the available data. Dose on the x-axis is the TiO_2_ lung dose (µg). Data of 6 exposure regimes are simultaneously fitted using covariate analysis. Blue downward triangle: 18 hours after single dosing (single1). Pink cross-plus: 7 days after single dosing (single7). Light blue cross-square: 14 days after single dosing (single14). Black triangle: 18 hours after repeated dosing (rep1). Red cross: 30 days after repeated dosing (rep30). Green diamond: 60 days after repeated dosing (rep60).

Due to the absence of a trend no BMD confidence interval is derived.

### TiO_2_, fraction PMN


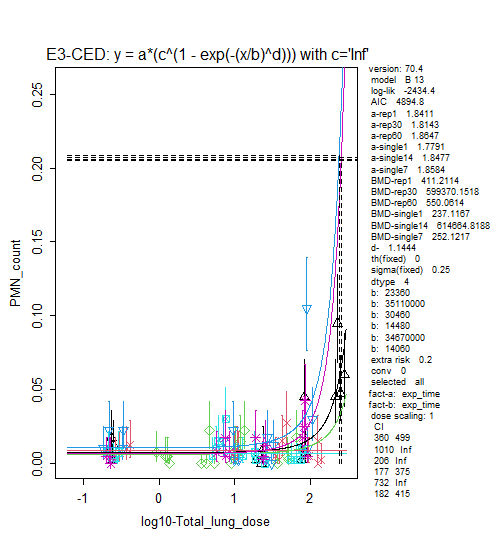


**Figure S11** Exponential dose-response model fitted to the PMN count in BALF (y-axis). Dose on the x-axis is the TiO_2_ lung dose (µg). Data of 6 exposure regimes are simultaneously fitted using covariate analysis. Blue downward triangle: 18 hours after single dosing (single1). Pink cross-plus: 7 days after single dosing (single7). Light blue cross-square: 14 days after single dosing (single14). Black triangle: 18 hours after repeated dosing (rep1). Red cross: 30 days after repeated dosing (rep30). Green diamond: 60 days after repeated dosing (rep60). Horizontal dashed line indicates the BMR at 20% extra risk. Vertical dashed lines indicate the BMDs of each exposure regime.


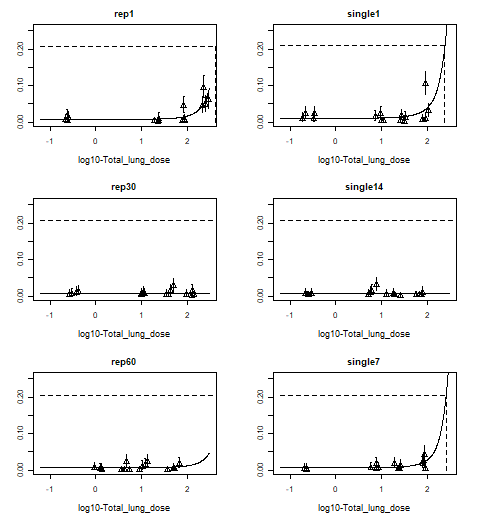


**Figure S12** Data and exponential model fits obtained in figure 11 now plotted separately for each exposure regime, indicated above each plot.

The BMD confidence interval is:

| subgroup | BMDL (µg) | BMDU (µg) |
| --- | --- | --- |
| rep1 (18 hours after repeated dosing) | 360 | 499 |
| rep30 (30 days after repeated dosing) | 1010 | Inf |
| rep60 (60 days after repeated dosing) | 206 | Inf |
| single1 (18 hours after single dosing) | 177 | 375 |
| single7 (7 days after single dosing) | 182 | 415 |
| single14 (14 days after single dosing) | 732 | Inf |

### TiO_2_, total protein


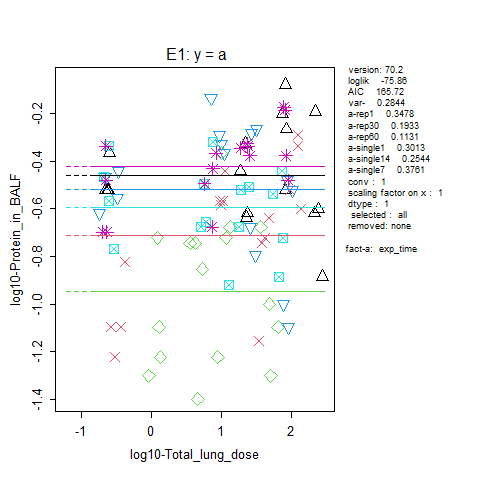


**Figure S13** The four continuous dose-response models (exponential, Hill, inverse exponential and lognormal) fitted to the total protein concentration in BALF (y-axis, g/L) all showed that there is no trend in the available data. Dose on the x-axis is the TiO_2_ lung dose (µg). Data of 6 exposure regimes are simultaneously fitted using covariate analysis. Blue downward triangle: 18 hours after single dosing (single1). Pink cross-plus: 7 days after single dosing (single7). Light blue cross-square: 14 days after single dosing (single14). Black triangle: 18 hours after repeated dosing (rep1). Red cross: 30 days after repeated dosing (rep30). Green diamond: 60 days after repeated dosing (rep60).

Due to the absence of a trend no BMD confidence interval is derived.
